# Metabolomics and genomics combine to unravel the pathway for the presence of fragrance in rice

**DOI:** 10.1038/s41598-017-07693-9

**Published:** 2017-08-18

**Authors:** Venea Dara Daygon, Mariafe Calingacion, Louise C. Forster, James J. De Voss, Brett D. Schwartz, Ben Ovenden, David E. Alonso, Susan R. McCouch, Mary J. Garson, Melissa A. Fitzgerald

**Affiliations:** 1 School of Agriculture and Food Sciences, The University of Queensland, St Lucia, QLD 4072 Australia; 2 School of Chemistry and Molecular Biosciences, The University of Queensland, St Lucia, QLD 4072 Australia; 3 NSW Department of Primary Industries, Yanco Agricultural Institute, Yanco, NSW 2703 Australia; 4 LECO Corporation Life Science and Chemical Analysis Centre, 1850 Hilltop Rd, Saint Joseph, MI 49085 USA; 5 Department of Plant Breeding & Genetics, Cornell University, Ithaca, NY 14853 USA

## Abstract

Since it was first characterised in 1983, 2-acetyl-1-pyrroline (2AP) has been considered to be the most important aroma compound in rice. In this study, we show four other amine heterocycles: 6-methyl, 5-oxo-2,3,4,5-tetrahydropyridine (6M5OTP), 2-acetylpyrrole, pyrrole and 1-pyrroline, that correlate strongly with the production of 2AP, and are present in consistent proportions in a set of elite aromatic rice varieties from South East Asia and Australia as well as in a collection of recombinant inbred lines (RILs) derived from *indica* Jasmine-type varieties, Australian long grain varieties (*temperate japonica*) and Basmati-type rice (*Grp V*). These compounds were detected through untargeted metabolite profiling by two-dimensional gas chromatography-time-of-flight mass spectrometry (GC × GC-TOF-MS), and their identity were confirmed by comparison with authentic standards analysed using gas chromatography mass spectrometry (GC-MS) and High Resolution GC × GC-TOF-MS (GC × GC HRT-4D). Genome-wide association analysis indicates that all compounds co-localised with a single quantitative trait locus (QTL) that harbours the *FGR* gene responsible for the production of GABA. Together, these data provide new insights into the production of 2AP, and evidence for understanding the pathway leading to the accumulation of aroma in fragrant rice.

## Introduction

Fragrant (or aromatic) rice commands the highest prices in the global rice market^1^. The trait of fragrance has been selected multiple times by ancient farmers throughout South and Southeast Asia, and multiple, independent alleles have been found in the same gene in different genetic backgrounds^2^. The strength of fragrance differs between varieties^3, 4^, and the most highly fragrant varieties tend to be the most popular among consumers^1^. For example, in Lao PDR, there is a fragrant rice variety called ‘Four Houses’, meaning that when this rice is cooking, its fragrance is enjoyed by people four houses away^5^. Emphasising strong fragrance through variety naming indicates the value that consumers place on highly fragrant rice.

Fragrance in rice is currently defined by a single compound, 1-(3,4-dihydro-2*H*-pyrrol-5-yl) ethanone, usually known by its synonym 2-acetyl-1-pyrroline (2AP)^4, 6^. Contrary to previous speculations that 2AP is produced in rice as a result of Maillard reaction^7^, it has been demonstrated that the presence of 2AP is due to loss-of-function mutations in the coding region of the *FGR* gene (Os08g0424500)^2, 8, 9^. Suppression of the *FGR* allele in non-aromatic rice varieties results in production of 2AP and leads to fragrance^9^, while transgenic expression of the *FGR* allele in *fgr*-carrying genotypes converts aromatic lines into non-aromatic lines^10^. These two studies conclusively confirm that alleles of the *FGR* gene are essential for the presence of 2AP in rice. Similarly, the inactivation of *FGR* orthologues in other plant species such as *Bassia latifolia* ^11^, soybean^12^, and cucumber^13^ also leads to the production of 2AP.

The *FGR* locus encodes an amino aldehyde dehydrogenase (AADH) which catalyses the oxidation of 4-aminobutanal, the product of oxidative deamination of putrescine^14^, to γ-aminobutyric acid (GABA)^9, 15^ as a stress response of plants^16^. Varieties carrying *fgr*, the recessive allele, produce a truncated AADH protein which cannot catalyse the oxidation of 4-aminobutanal to GABA^15^. It has been reported that 4-aminobutanal accumulates, and cyclisation and dehydration result in the formation of 1-pyrroline, which becomes acetylated to form 2AP^10, 11, 15^. It is also widely proposed that 1-pyrroline is directly acetylated to 2AP by methylglyoxal^17, 18^ or acetyl CoA^15, 19^. Moreover, reports have demonstrated that the addition of 1-pyrroline to *Bacillus aureus* culture medium^20^ and to unheated rice callus^21^ leads to a significant increase in 2AP, although in these studies, the recovery of 2AP is low. Together, these studies suggest firstly that 1-pyrroline (or a biological equivalent) could participate in the pathway leading to 2AP; and secondly that the temperature sensitivity of 2AP formation implies an enzyme-mediated synthesis.

Previous analyses of the volatile compounds in rice have consistently detected an unknown compound in fragrant rice varieties^22–24^. The similarity of the mass spectral electron ionisation (EI) fragmentation pattern of the unknown compound to that of 2AP has led to suggestions that the compound is an isomer of 2AP^23, 24^. However, both the identity of the compound, and any role it plays in 2AP synthesis, are yet to be determined. There remains, therefore, much to understand about the processes regulating the accumulation of 2AP in fragrant rice grains.

The objectives of the present paper, using a collection of advanced breeding lines developed from diverse rice varieties, were to (i) perform metabolite profiling using several analytical platforms to identify metabolites in the pathway of 2AP synthesis; (ii) conduct genome-wide association studies (GWAS) to identify Quantitative Trait Loci (QTL) associated with the accumulation of relevant metabolites; and (iii) further understand the pathway leading to aroma in rice.

## Results

### Untargeted metabolite profiling of volatile compounds

**Figure 1:**
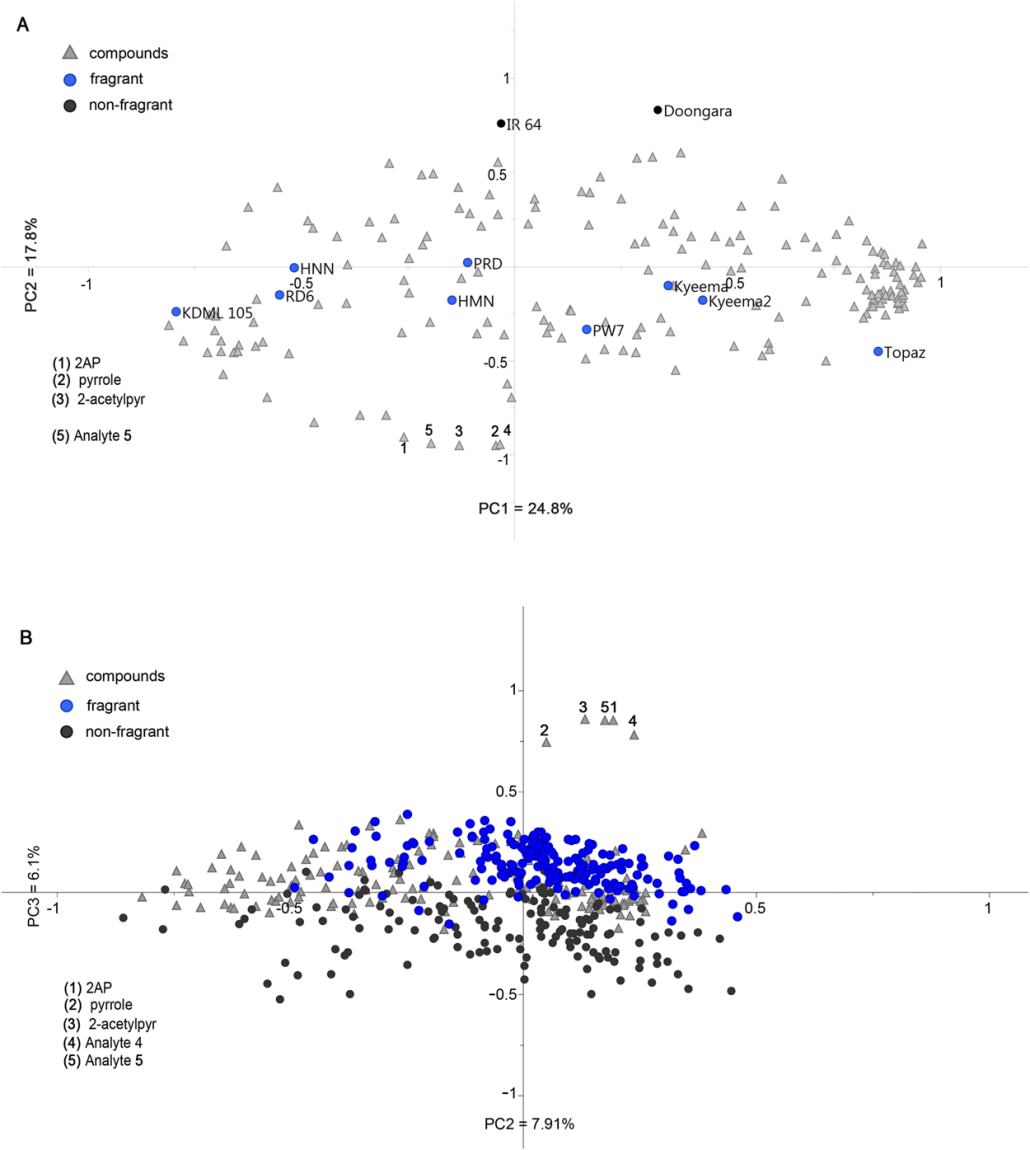
PCA biplot of the volatile metabolites detected in rice by GC × GC-TOF-MS. Metabolites are the triangles, overlayed with the rice varieties (circles). (**A**) Set 1 (n = 11), PC1 = 24.8% and PC2 = 17.8%. (**B**) Set 2 (n = 341) PC2 = 7.91% and PC3 = 6.1%.

To identify metabolites that relate to the pathway of 2AP, we performed metabolite profiling of volatile compounds in a test set of 10 commercial rice varieties using GC × GC-TOF-MS. A total of 175 putative compounds were detected and were used for multivariate analysis (Fig. 1A). The principal components analysis (PCA) biplot shows that 24.8% of total variation is explained by PC1. The samples separated according to germplasm class (*indica* vs *japonica* varieties) along PC1, with the *indica* samples clustering in the left of the axis and the *japonica* varieties on the right of the axis, with one exception. KDML 105, a Thai *indica* variety, grouped with the *japonica* samples, as was also observed in our previous study^22^ using a similar set of varieties. A clear separation between the fragrant and the non-fragrant rices was observed along PC2, which explains 17.8% of total variation. To determine which of the compounds are the most discriminating between aromatic and nonaromatic rice, OPLS-DA was conducted (Figure S1). The five compounds with the highest variable importance in projection (VIP) scores in the aromatic cluster were: 2-acetyl-1-pyrroline (Analyte **1**), pyrrole (Analyte **2**), 2-acetylpyrrole (Analyte **3**), Analyte **4** and Analyte **5**. Analytes **1**, **2**, and **3** had convincingly high similarity matches to the library (NIST 11 v2.0) mass spectra (94%, 90.9% and 94.5%, respectively) and the annotations were subsequently validated against analytical standards (2AP synthesised in this study; pyrrole, 98% and 2-acetylpyrrole, 99% both Sigma-Aldrich, MO, USA) (Figure S2). Analytes **4** and **5** (identified as 1-ethenylaziridine and 1-piperidine by the library search) were treated as unannotated compounds due to the relatively low similarity matches of 81.1% and 77.7% respectively. The mass spectral data of **4:** *m*/*z* (%) 69 (48), 68 (24), 54 (2), 42 (27), 41 (100), 40 (17), 39 (22), 38 (11); and **5:** 111 (10), 84 (2), 83 (17), 66 (2), 55 (100), 54 (14), 52 (6), 42 (84), 41 (30), 40 (14), 39 (33) matched the library spectra poorly. The spectral data and retention time of Analyte **5** matches that of an unknown compound (Analyte 9) in our previous study^22^. A correlation analysis between the five compounds detected in the aromatic rices shows a strong correlation between 2AP and 2-acetylpyrrole (R^2^ = 0.99, Table 1), 2AP and Analyte **5** (R^2^ = 1), and 2-acetylpyrrole and Analyte **5** (R^2^ = 0.99). A weak correlation was found between Analyte **4** with all the compounds except for pyrrole (R^2^ = 0.78).

**Table 1:** Correlation coefficients of 2AP linked compounds in Set 1 (n = 11) and Set 2 (n = 341).

Set 1	(1)	(2)	(3)	(4)	(5)
2-acetyl-1-pyrroline (**1**)	1				
pyrrole (**2**)	0.68 (*0*.*04*)	1			
2-acetylpyrrole (**3**)	0.99 (<*0*.*0001*)	0.64 *0*.*06*	1		
Analyte **4**	0.1 *0*.*79*	0.78 *0*.*01*	0.08 *0*.*84*	1	
Analyte **5**	1 (<*0*.*0001*)	0.66 *0*.*05*	0.99 (<*0*.*0001*)	0.09 *0*.*83*	1
**Set 2**					
2-acetyl-1-pyrroline (**1**)	1				
pyrrole (**2**)	0.62 (<*0*.*0001*)	1			
2-acetylpyrrole (**3**)	0.61 (<*0*.*0001*)	0.85 (<*0*.*0001*)	1		
Analyte **4**	0.47 (<*0*.*0001*)	0.65 (<*0*.*0001*)	0.56 (<*0*.*0001*)	1	
Analyte **5**	0.98 (<*0*.*0001*)	0.62 (<*0*.*0001*)	0.62 (<*0*.*0001*)	0.44 (<*0*.*0001*)	1

*P* values are given in italics.

A collection of 341 rice samples (Set 2), planted in a different season and location (see Methods section), was used to confirm the metabolites detected on the elite commercial samples. PCA showed that 2AP and the linked compounds discriminate between fragrant and non-fragrant rice samples in the third PC, which explains 6.1% of total variation observed (Fig. 1B). Furthermore, the strong correlations between the five compounds were once again confirmed (Table 1). There was a strong correlation between 2AP and Analyte **5**, while the observed positive correlation between 2AP and 2-acetylpyrrole was seen to decrease in the bigger set (R^2^ = 0.61 from R^2^ = 0.99). The strength of the correlation between Analyte **4** and the other compounds generally increased in Set 2, although the highest association was only 0.65, again with pyrrole. Next, the relative amounts of the five compounds were compared between both sets of samples. A full model, accounting for sample group and compound, was fit to the data. In the usual fashion, a second, nested model, which omitted sample group as predictor variable, was fit, and the two models compared. There was no significant loss of fit to the data by removal of the sample group as predictor, indicating that variation between the two groups was not significant (χ^2^ = 7.3193, df = 5, p = 0.198). The proportion of 2AP to Analyte **5** was calculated in the fragrant accessions of Set 2, and an apparent 4:1 ratio was consistently noted (0.79:0.21 with a standard deviation of 0.05, Table S1). These analyses confirm the linkage between all five compounds, and emphasised the need to further characterise and identify Analytes **4** and **5**.

### Identification of unknown compounds

#### Analyte 4

Using high resolution MS (EI) analysis by HRT 4D, Analyte **4** had ions at *m*/*z* C_2_H_3_N 41.0260, C_4_H_6_N 68.0497 and C_4_H_7_N 69.0573 (Figure S3A) with mass accuracies of 0.01, 0.59 and −0.01 ppm, respectively. The molecular ion corresponds to a molecular formula of C_4_H_7_N (calc: 69.0578), which is expected for 1-pyrroline, the putative precursor in the biosynthesis of 2AP^15^. The reported EI mass spectral fragmentation pattern of 1-pyrroline *m*/*z* (%) (69 (68), 68 (31), 42 (41), 41 (100))^25, 26^ is similar to that observed, and therefore 1-pyrroline became a logical candidate for the identity of Analyte **4**. We therefore synthesised an authentic sample of 1-pyrroline using literature methods^26^ and found that it possessed identical mass spectrometric properties (Figure S4A,B) and spectroscopic (Proton nuclear magnetic resonance,^1^H NMR, Figure S4C) to those reported in the literature^25, 26^. When analysed by GC × GC TOF-MS, the retention time of 1-pyrroline was identical to that of Analyte **4**, at 370 s and 0.7 s in the primary and the secondary columns, respectively. The MS EI fragmentation of Analyte **4** (*m*/*z* (%) 69 (48), 68 (30), 41(100)) detected in rice, matched that of the synthetic sample (Figure S4), confirming the identification of 1-pyrroline.

#### Analyte 5

HRT 4D analysis of Analyte **5** shows ions at *m*/*z* C_2_H_4_N 42.0463, C_3_H_5_N 55.0416, C_5_H_9_N 83.0729 and a molecular ion of C_6_H_9_NO 111.0680, with mass accuracies of −0.01, 1.21, −0.61 and −0.92 ppm, respectively (Figure S3B). High resolution MS analysis (CI) showed an accurate mass value of 112.07572 [MH]^+^ which correlated to a molecular formula of C_6_H_10_NO, with mass accuracy of 0.29 ppm. Given that it was unconvincingly annotated by the mass spectral library search, and since C_6_H_9_NO is the same formula as that of 2AP, we considered an isomeric structure of 2AP as the candidate for Analyte **5**.

**Figure 2:**
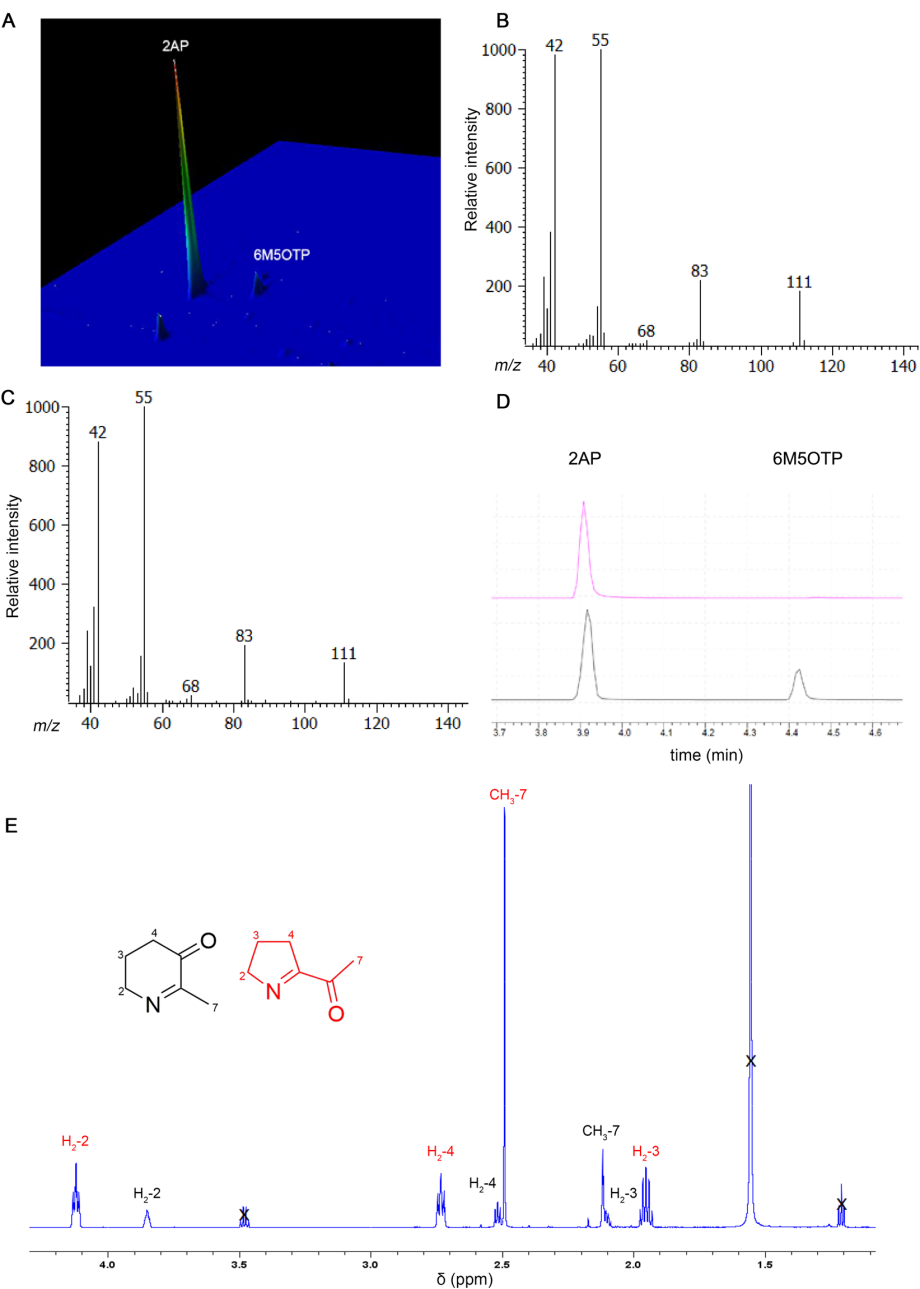
Identification of Analyte 5 as (6M5OTP). (**A**) GC × GC-TOF-MS 3D chromatogram of synthesised 2AP standard showing 2AP (RT: 627.5 s, 1.025 s) and 6M5OTP (RT: 707.5 s, 1.27 s). MS Electron ionisation fragmentation pattern of Analyte 5 detected by GC × GC-TOF-MS (**B**) synthesised 2 AP standard, and (**C**) rice. (**D**) GC-MS trace comparison of ratio between 2AP and 6M5OTP before (above) and after acid-base extraction (below). (**E**) Expansion of the^1^H NMR of 2AP (red) and 6M5OTP (black). Solvent peaks are denoted in X.

We synthesised authentic 2AP standards (Supplementary Information S1), which produced a mixture of 2AP and a second compound (Fig. 2A). When analysed by GC × GC TOF-MS, the retention time (707.5 s, 1.2 s) and mass spectral fragmentation pattern of the second compound (Fig. 2B) is identical to that of Analyte **5** detected in rice (Fig. 2C). Furthermore, 2AP and the second compound was found to be in a 99:1 ratio by GC - MS. Subjecting the reaction product to a single round of acid-base partitioning increased the concentration of the second compound to a ratio of 76:24 (GC-MS) (Fig. 2D). A second acid-base extraction further increased the product ratio to approximately 73:27 (GC-MS). The^1^H NMR spectrum of the second compound (^1^H NMR: δ 3.78–3.85 (m, 2H), 2.48 (t, *J* = 6.9 Hz, 2H), 2.08 (s, 3H), 2.03–2.10 (m, 2H), Fig. 2E) matched that reported by De Kimpe^27^, and by Harrison and Dake^28^ for 6-methyl-5-oxo-2,3,4,5-tetrahydropyridine, as did the^13^C NMR data obtained from 2D (HSQC, HMBC) NMR spectra (Figure S5). Taken together, these data all indicate that Analyte **5** is 6-methyl-5-oxo-2,3,4,5-tetrahydropyridine (6M5OTP), an isomer of 2AP.

### GWA analysis for 2AP and linked compounds

The milled rice grains from a collection of 341 rice lines was phenotyped to determine the relative concentrations of the five volatile compounds in each line (as described above) and the same plant was genotyped to obtain a genetic profile for each line. Using Genotyping-by-Sequencing (GBS), a total of 233,214,413 short sequencing reads were produced from the 384 barcoded samples, with an average number of reads per individual of 572,545. Only one sample (Y20140298) generated <10% of the mean barcode reads value (MBRV) and was discarded, while blank samples generated <1% of the total MBRV. 1,888,334 tags were aligned with the Nipponbare reference genome (MSU version 7.0) and 87.6% aligned to unique positions. A total of 240,250 Single Nucleotide Polymorphisms (SNPs) were discovered in the initial allele calling. SNPs were filtered on call rates (>=75%) and minimum allele frequency (>=0.05), resulting in a dataset consisting of 33,725 SNPs that were well-distributed across the genome. This SNP matrix was used for further data analysis and association mapping.

**Figure 3:**
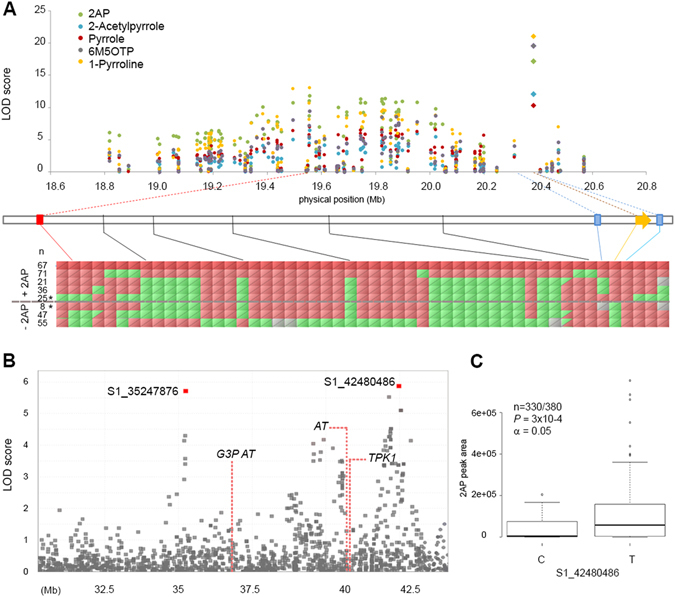
GWAS of 2AP and linked compounds. (**A**) Physical distribution of the most significant SNPs in chromosome 8. The functional markers for *FGR* (diamonds) outperformed the MS SNPs (S8_19555833) for each of the five compounds. Lines connecting (**A**,**B**) denote the position of the S8_19555833 (previously MS SNP, red line) and the SNPs closest to *FGR* (SNPs S8_20311425 and S8_20408314, blue lines) relative to *FGR* (orange arrow). (**B**) Representative haplotypes of the population. Red cells represent SNPs identical to the aromatic line KDML 105, heterozygotes are represented by half-filled cells, while grey are missing data. The number of lines carrying each haplotype is indicated in the table. Asterisk denotes the haplotypes where linkage breaks between the MS SNP S8_19555833 are not predictive of the presence or absence of aroma, explaining the lower LOD score of SNP S8_19555833. (**C**) Zoom in of the QTL region on chromosome 1. The most significant SNPs (MS SNPs) are denoted in red. Location of the candidate genes are shown by dotted lines. (**D**) Box plot for the 2AP levels at different alleles of the MS SNP S1_42480486. The middle line indicates the median, the box indicates the range of the 25th to 75th percentiles of the total data, the whiskers indicate the interquartile range and the outer dots are outliers. *n* shows the number of samples used to make the plot over the total number of samples, *p* and *α* denote the p-values and alpha, respectively.

For GWAS on all 340 lines, a large QTL for 2AP was identified on chromosome 8 (logarithm of odds, LOD score = 14) near the *FGR* locus^8^. Co-located QTLs were found in the same region for the presence of 2-acetylpyrrole, pyrrole, 1-pyrroline and 6M5OTP (LOD scores of = 10.7, 9.3, 16.7 and 16.6, respectively) (Fig. 3A, Figure S6). The Manhattan and QQ plots for this GWAS are given in Figure S6. The most significant SNP (MS SNP) S8_19555833 (LOD score = 14) mapped 750 kb (~3 cM) away from the *FGR* locus (Fig. 3). No SNP markers from the GBS dataset mapped within the coding region of *FGR*. Two SNPs – S8_20311425 and S8_20408314 - were found to be closer to *FGR* (68 kb and 29 kb from the translational start site, respectively), but both were present at low frequency and did not segregate with the phenotype. To test the hypothesis that polymorphism in the *FGR* gene might be responsible for the variation observed in the levels of the five compounds, 262 of the lines were further screened using allele-specific “functional” markers for *FGR* targeting 8 bp indel and 3 SNPs in seventh exon of the *FGR* gene^29^. Ninety seven progenies were found to carry the functional form of the gene (*Fgr* allele conferring no fragrance) while 165 carried the recessive (mutated) version (*fgr* allele conferring fragrance), and the marker perfectly predicted the phenotype (Table S1, Fig. 3B). Upon addition of the *FGR* functional marker as a covariate to the GWAS model, the peak shifted toward the *FGR* gene for all five compounds, and the significance of the functional marker surpassed that of SNP S8_19555833 (Fig. 3A).

A second, minor-effect QTL for levels of 2AP was observed on chromosome 1 (Figure S6A). Further examination of this region revealed two QTL peaks, with MS SNPs S1_35247876 (LOD score = 6.06) and S1_42480486 (LOD score = 5.96), approximately 7 Mb apart (Fig. 3C). SNP S1_35247876 has a slightly higher LOD score than S1_42480486, however, the former also has more ‘N’ genotypes or missing values (72 versus 10 missing genotypes, respectively). Therefore we used S1_42480486 in looking at the contribution of the different alleles (C/T) to the levels of 2AP (Fig. 3D). Samples carrying a ‘T’ SNP show significantly more 2AP production (p < 0.0003) compared to those carrying a ‘C’ SNP. Scanning the region between the two MS SNPs, we found several genes which are directly or indirectly involved in the transfer of either an acyl group or a two-carbon subunit, such as *thiamine pyrophosphate kinase 1* (*TPK1*, OS01G0931400, LOC_Os01g70580 start site 1: 40871884), *acyltransferase* (*AT*, OS01g0931300, LOC_Os01g70570 start site 1:40868356), and *glycerol-3-phosphate acyltransferase* (*G3PAT*, Os01g0855000, LOC_Os01g63580 start site 1:36866375) (Fig. 3C). While none of these genes are in strong linkage disequilibrium (LD) with the MS SNPs examined here, it would be worth investigating whether allelic variation at one or more of these candidate genes may impact levels of 2AP.

## Discussion

Previous studies^2, 8, 23, 24^ on rice aroma have focused on 2AP as the major aromatic compound. Here, we present four other compounds: pyrrole, 2-acetylpyrrole 1-pyrroline, and 6-methyl-5-oxo-2,3,4,5-tetrahydropyridine (6M5OTP) that are highly discriminating between aromatic and non-aromatic rices, and which associate strongly with 2AP, both chemically and genetically (Fig. 1). The genetic and metabolomic data presented here indicate strongly that these five compounds all participate in one pathway. Two of the compounds, 6M5OTP and 1-pyrroline, are identified here for the first time in rice. The compound 6M5OTP appears to have been previously detected in other aromatic rices^22–24^ but never correctly identified. The compound 1-pyrroline has long been considered the precursor of 2AP, but has not been previously detected in rice. In the past, the floral, popcorn and toast smell of rice has been attributed to 2AP alone. However, its structural isomer 6M5OTP has been reported to have a similar scent perceivable through gas chromatography olfactory analysis^24^. It is now evident from our chemical study that 2AP co-occurs with 6M5OTP. This means that 6M5OTP, presumably produced concomitantly with 2AP in rice, contributes to the distinguishing aroma of fragrant rice.

Strong correlations were found between 2AP, 2-acetylpyrrole and 6M5OTP in the Asian and Australian elite aromatic varieties; these were also observed in the larger collection grown in a different location and experimental season (Table 1), suggesting that this occurrence could be universal to all aromatic rices. 2AP can easily oxidise to 2-acetylpyrrole at room temperature^30^, which could explain the correlation between these two compounds. The same oxidation process is likely to explain the correlation between 1-pyrroline and pyrrole^31^. Both 2AP and 2-acetylpyrrole have sweet aroma reminiscent of cooked popcorn scent^6^. However, the odour threshold of 2-acetyl pyrrole is 170000 ppb as compared to 2AP (0.06 ppb)^6^, suggesting that the oxidation of 2AP leads to loss of favourable aroma in rice.

The major QTL peak for all five heterocycles localised in the region of the *FGR* gene. This could be explained by several hypotheses: (i) there is allelic variation for a master switch that pleiotropically governs the accumulation of multiple compounds; (ii) there is allelic variation for a gene that governs the expression of a critical node in one pathway, and variation in that pathway indirectly affects other pathways that determine the accumulation of the other compounds; (iii) there is an array of linked genes within the QTL peak. To test for the possibility of a master switch, the population was screened for the major *FGR* allele^2^ using previously developed markers^29^. Upon rerun of the GWAS, the functional *FGR* marker was found to be more significantly associated with both fragrance and levels of all 5 compounds than the MS SNP (S8_19555833). To understand whether this was due to recombination separating S8_19555833 from the putative functional gene, *FGR*, we examined the genotypic profiles and identified 33 recombinants between S8_19555833 and the functional *FGR* marker (Fig. 3A). This explained the lower level of significance for the genotype-phenotype association, and supported the conclusion that the loss of function of *FGR* is indeed the master switch that determines the increase in levels of all five compounds.

This study shows, for the first time in rice, that 1-pyrroline is a volatile compound that is detected only in fragrant rice (Fig. 1A). If 1-pyrroline is the immediate precursor of 2AP, it would be expected to correlate strongly with 2AP. However, this study shows that the correlations between 1-pyrroline and either 2AP or 6M5OTP are the weakest amongst the compounds (Table 1). Likewise, previous attempts to synthesise 2AP *in vivo* and *in vitro* from 1-pyrroline gave very poor yields. *In vitro* synthesis of 2AP from 1-pyrroline-5-carboxylic acid and methylglyoxal yielded only 6.3%^32^, and a similar synthesis of a 2AP analogue from phenylglyoxal gave a 3.7% recovery^26^. *In vivo*, addition of 1-pyrroline to non-fragrant rice at physiological pH has the highest yield of 0.05% conversion to 2AP in unheated callus at pH 8.5^21^. These studies indicate that acceptable amounts of 2AP do not form *in vitro* or in the callus at physiological pH from 1-pyrroline. These previous reports, together with the weak correlation between 2AP and 1-pyrroline reported here, suggest that direct acetylation of 1-pyrroline may not be the primary pathway for the formation of 2AP.

**Figure 4:**
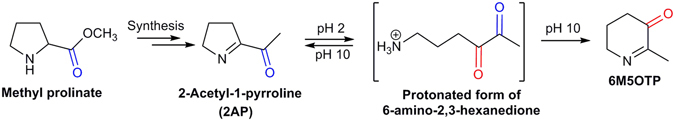
Chemical formation of 6M5OTP through *in vitro* acid/base workup. 2AP ring opens in excess acid to form an unstable diketone, which cyclises back to 2AP or to 6M5OTP under basic conditions.

The present paper, as well as several previous studies^27, 28^ supports the possibility of ring opening *in vitro* when 2AP is subjected to acidic conditions. De Kimpe *et al*.^27^ isolated 6M5OTP as a minor product during acid-catalysed deprotection of imidoyl- or diethoxyethyl-pyrrolines. Likewise, during deprotection of *N*-Boc-protected pyrroline, Harrison and Dake^28^ reported the isolation of 6M5OTP. In the present study, an acid-base workup of the synthesised 2AP led to an increase in the proportion of 6M5OTP, indicating ring opening of 2AP followed by ring closure. The ring opening of 2AP leads to an unstable, linear diketone intermediate – 6-amino-2,3-hexanedione (Fig. 4), which differentially cyclises at carbon 2 (C2) or at C3 resulting in 2AP or 6M5OTP, respectively, when the reaction solution is made basic. These experiments demonstrated the importance of the formation of the diketone intermediate in the production of 2AP and 6M5OTP.

The clear chemical and genetic linkage between 6M5OTP and 2AP raises questions about the biosynthesis of 2AP. It is well known that 4-aminobutanal is derived from putrescine in the pathway of polyamine oxidation to GABA, and in rice, this process occurs only in the peroxisome^33, 34^. The transfer of two carbon units, such as is required to form 2AP from 4-aminobutanal or 1-pyrroline, has been observed in several biochemical processes, such as the fatty acid ß-oxidation producing acetyl CoA, the non-oxidative stages of the pentose phosphate pathway in the cytoplasm, and the synthesis of diacetyl and acetoin in microbes. Previous reports have speculated that acetyl CoA could be a source of the acetyl group in 2AP^15, 20^. However, the biochemical role of acetyl CoA is usually associated with β-keto thioester formation (eg. fatty acid biosynthesis) rather than α-imino ketone or 1,2-diketone formation, as is required in 2AP biosynthesis, and therefore acetyl CoA is not necessarily the source of the two carbon units of 2AP. Previous precursor studies implicated acetaldehyde (from ethanol^19^) or pyruvate^35^ as the most likely donor for the acetyl group of 2AP.

**Figure 5:**
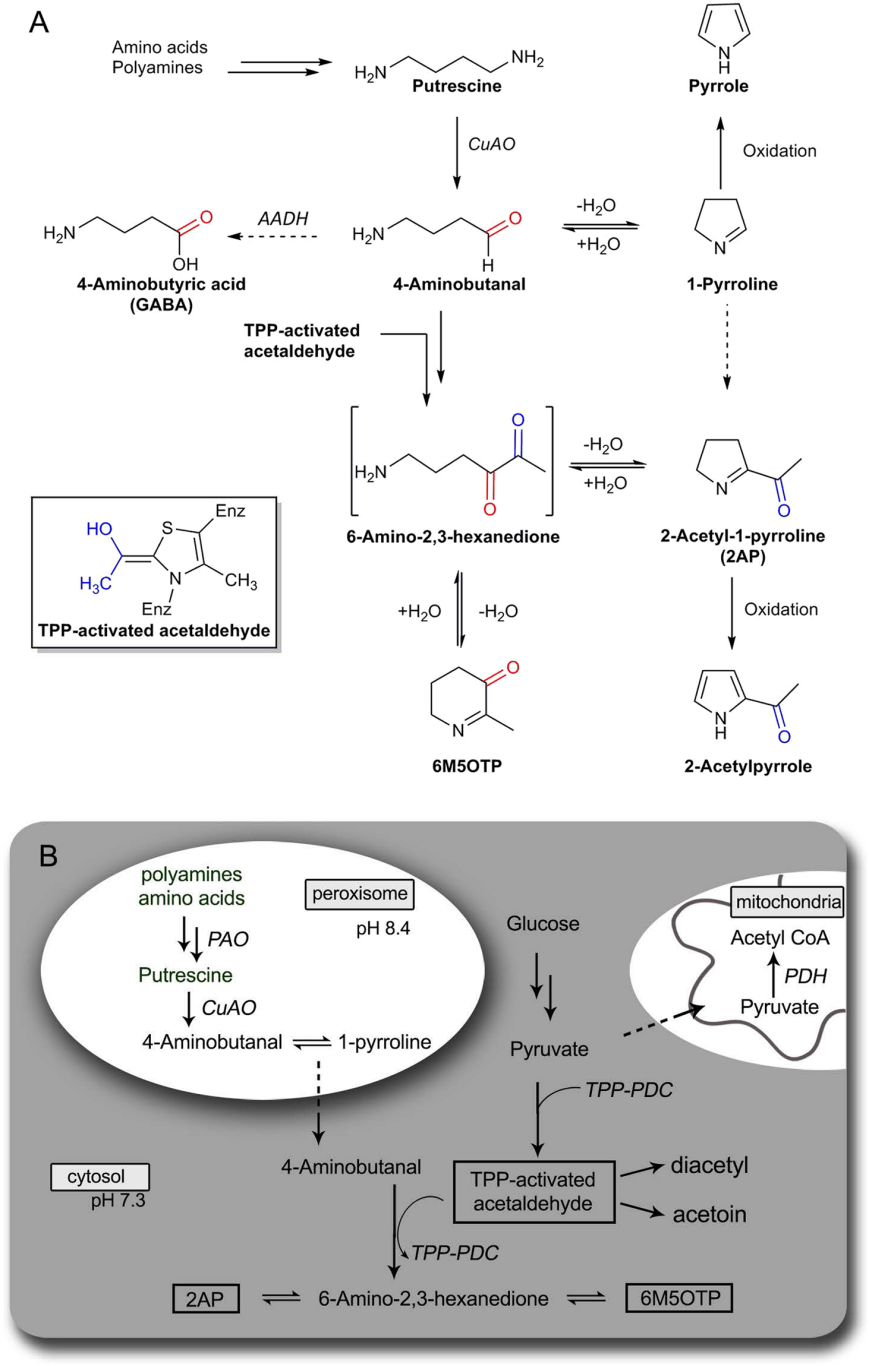
Putative biosynthesis of 2AP. Enzymes are in italics: CuAO – copper amine oxidase; PAO – polyamine oxidase; AADH – amino aldehyde dehydrogenase; PDC – Pyruvate decarboxylase; PDH – pyruvate dehydrogenase complex. (**A**) TPP-catalysed transfer of a C2 unit to 4-aminobutanal. The reaction with 4-aminobutanal forms an unstable diketone 6-amino2,3-hexadione, which cyclises to either 2AP or 6M5OTP. (**B**) Cellular localisation of the reactions leading to the formation of 2AP and 6M5OTP.

Mechanistic considerations suggest that the pathway for the transfer of two carbon units to 4-aminobutanal could be similar to the way acetoin and diacetyl are biosynthesised^36, 37^. The production of these from pyruvate is initiated by the pyruvate dehydrogenase complex or pyruvate decarboxylase^38, 39^ utilising a cofactor - thiamine diphosphate, formerly called thiamine pyrophosphate (TPP). In these reactions, the thiazolium ring of TPP is involved in the transfer of two carbon units via an hydroxyethyl-TPP enzyme complex^40, 41^ that corresponds to an “activated acetaldehyde” unit. This process has been shown to occur in developing pea seeds^36^ and wheat grains^37^. The enzymes required to synthesise acetoin are expressed in developing rice grains^42, 43^, and acetoin is one of the compounds found in grains in the present study (Table S1). Therefore, following the mechanistic action of the TPP-catalysed formation of diacetyl and acetoin, 2AP synthesis in rice could be occurring as follows (Fig. 5A). The “active acetaldehyde” could react with the carbonyl of 4-aminobutanal. Reaction of the aldehyde produces a hydroxyethyl intermediate, which would rapidly oxidise to the diketone 6-amino-2,3-hexanedione. The primary amine of the dione would then attack either of the carbonyl functions of the diketone moiety to produce either 2AP, when the ring closes at C3, or if the ring closes at C2, 6M5OTP would be formed. Further studies would be useful to replicate previous attempts to synthesise 2AP from 1-pyrroline^21, 26^ in the presence of TPP.

Pyruvate is produced by glycolysis in the cytoplasm. It is then transported into mitochondria and oxidised by the pyruvate dehydrogenase complex, forming acetyl CoA^44^. It can also be decarboxylated by pyruvate decarboxylase (PDC) in the cytoplasm^45^. Both enzymes require TPP, which is ubiquitous^46^. If pyruvate is decarboxylated to form active acetaldehyde, it must be proximal to 4-aminobutanal or 1-pyrroline in order to react and form 2AP. In non-fragrant rice, the GABA produced from 4-aminobutanal exits the peroxisome^34^ through a channel-forming protein that allows small molecules, below 400 Da, to cross the peroxisomal membrane passively^33, 47^. In fragrant rice, the channel-forming protein may allow 4-aminobutanal or 1-pyrroline to exit the peroxisome into the cytosol in the same way as other small molecules, where a reaction with activated acetaldehyde from PDC could lead to the production of 2AP and 6M5OTP (Fig. 5B). At physiological pH, 4-aminobutanal is a minor component (<5%) of a complex equilibrium, which contains several other chemical species, including a hydrate, a hemiaminal and the cyclic 1-pyrroline^48^. While acetylation of 1-pyrroline could, in theory, lead directly to 2AP, the formation of 6M5OTP under physiological conditions requires the presence of the linear dicarbonyl, suggesting formation from the aldehyde. Future studies to confirm the movement of GABA, 4-aminobutanal or 1-pyrroline from the peroxisome to the cytoplasm are underway.

Minor effect QTLs were detected for the presence of 2AP on chromosome 1 (Fig. 3C). Candidate genes within this region are *TPK1*, *G3PAT* and *AT*. The two latter genes, however, are involved in the transfer of long chain acyl groups (greater than C10^49^), therefore would be less likely to be involved in the production of 2AP. *TPK1* encodes thiamine pyrophosphate kinase, responsible for catalysing the conversion of thiamine to TPP, and thus is a more probable candidate gene in terms of function, and is consistent with the likely pathway of acetylation (Fig. 5B). The presence of regulatory motifs in the upstream or downstream regions of this gene may also contribute to different levels of fragrance in rice, a hypothesis that could be explored by further sequencing and molecular assays. Nonetheless, new insights into the genetic and biochemical route of 2AP production would enable breeders to select more accurately for highly fragrant rice.

## Methodology

### Rice materials

The first set of samples (Set 1) consisted of seven aromatic and two non-aromatic varieties of rice (Table 2). The samples were sown in Mackay Queensland, in the summer season from December 2013, and harvested in April 2014. A second set of samples (Set 2) consisted of an association mapping population comprised of multiple families derived from crosses between the varieties in Set 1 and elite Australian long grain varieties, giving a diverse collection of 380 genotypes (Table S1). These were grown as F7 RILs at the Leeton Field Station (Yanco, NSW), the Australian Rice Breeding Program between November 2013 and April 2014. Field management, irrigation, pesticide and fertiliser treatments all followed typical commercial practices. Single plants were tagged, a single leaf from each was preserved for DNA extraction and genotyping, and the grain from two panicles was collected for metabolomics profiling. Out of the 380 samples, 39 did not produced enough grains for metabolomic profiling, or had unfilled panicles due to photoperiod and/or cold sensitivity, giving a final number of 341 samples. All panicles were dried to 14% moisture and then the grain was shipped to The University of Queensland as paddy to minimise any possible release of volatile compounds or potential oxidation of lipids by damaging the bran. The paddy rices were dehulled using an Otake dehuller (Model FC2K, Aichi, Japan), and polished with a Kett Mill (Pearlest Grain Polisher, Tokyo, Japan). The white rices were cryoground to fine powder using a Geno Grinder (2010; Stanmore, UK). Rice flour was stored at −80 °C until analysis.

**Table 2:** Elite rice varieties from Southeast Asia and Australia used in this study (Set 1).

Variety	Germplasm Class	Origin	Phenotype	*FGR* genotype
Khao Khor 6 (RD 6)	Indica	Thailand	Fragrant	*fgr*
Hom Mali Niaw (HMN)*	Indica	Thailand	Fragrant	*fgr*
Khao Dawk Mali 105 (KDML105)	Indica	Thailand	Fragrant	*fgr*
Hom Nang Neuang (HNN)	Indica	Laos	Fragrant	*fgr*
Phka Rum Duol (PRD)	Indica	Cambodia	Fragrant	*fgr*
IR64	Indica	Philippines	Non- fragrant	*Fgr*
Doongara	Temperate Japonica	Australia	Non- fragrant	*Fgr*
Kyeema*	Temperate Japonica	Australia	Fragrant	*fgr*
Pandan Wangi 7	Tropical Japonica	Indonesia	Fragrant	*fgr*

*Two samples from different plots have been used in Set 1.

### Metabolite profiling

#### Comprehensive GC × GC-TOF-MS

Volatile compounds in all of the rice samples (Sets 1 and 2) were measured on one gram of rice flour by static headspace extraction using GC × GC-TOF-MS (Pegasus 4D, LECO Corporation; St. Joseph, MI, USA) following previously described protocols^22^. The full details of the metabolite profiling including GC × GC program settings, MS conditions and chromatogram alignment parameters are given in Table S2 and Supplementary Information S2. Quality control standards (commercial rice flour) were injected every 10 samples to monitor the performance of the instrument and to check for batch effects. Data preprocessing, baseline correction and identification of putative compounds were done using the software LECO ChromaTOF 4.50 with library match searching to Nist 11 v2.0. Commercial standards of several heterocycles, alcohols, ketones and aldehydes were either purchased or synthesized and used for retention time, identification of compounds, and confirmation of library annotation. All samples were analysed in two technical replicates.

#### High Resolution GC × GC-TOF-MS (GC × GC-HRT 4D)

Five rice samples exhibiting high levels of 2AP were analysed with a Pegasus HRT 4D at LECO Life Science and Chemical Analysis Centre Michigan, USA. The acquisition parameters were identical to those reported above. The mass spectrometer was set to electron ionisation (EI) mode to enable investigation of the fragmentation pattern of the compounds and allow comparison against the NIST library. Samples were further analysed using chemical ionisation (CI) detection to preserve the molecular ion and to identify the accurate mass of the compound. The acquisition mode was at high resolution R = 25,000 full width at half maximum (FWHM). The mass spectra were recorded at a mass range of 35–400 *m*/*z* and an acquisition rate of 200 spectra/s.

### Synthesis of authentic standards

In order to understand further the relationship between 2AP and analytes that associated with it in rice samples, 2AP and 1-pyrroline were both synthesised from proline^26, 50, 51^. For 2AP, the carbomethoxy substitutent of methyl prolinate was converted into an acetyl group by (i) *N*-chlorination followed by elimination of HCl using Et_3_N; and (ii) addition of MeMgBr in anhydrous Et_2_O at 0 °C followed by reaction workup using aqueous NH_4_Cl; (iii) purification by flash chromatography on base-washed silica using diethyl ether: pentane (1:3) as eluent. For 1-pyrroline, proline was oxidised with sodium metaperiodate and the resulting product purified by alumina chromatography using *n-*pentane:diethyl ether (8:2) as eluent. The full details of each synthesis are given in Supplementary Information S1.

The behaviour of the synthesised 2AP upon exposure to acid-base conditions was examined by both GC-MS (Shimadzu GC-MS-QP2010) and^1^H NMR (Bruker Avance DRX 700 spectrometer). A synthetic sample of 2AP was subjected to two rounds of acid-base partitioning by extraction into 1M HCl, followed by basification to pH 10, and extraction into chloroform. The accompanying GC-MS and^1^H NMR data are given in Supplementary Information S2.

### Genotyping

#### Genotyping-By-Sequencing (GBS)

Total genomic DNA was extracted from freeze dried leaf materials using Qiagen 96-plex DNEasy Plant DNA Extraction kit (Qiagen Hilden, Germany) following the manufacturer’s protocol. The DNA was quantified to 50–100 ng/ul using a Qubit 2.0 fluorometer (Invitrogen, USA). DNA quality was tested by digesting 500 ng DNA with 5 units of methylation insensitive restriction enzyme *Hind*III. The digested and uncut DNA samples were run on 0.8–1% agarose gel using λ *Hind*III as size standard. High-quality DNA samples that showed complete digestion on the gel were submitted for GBS.

The 384-plex GBS libraries were prepared at the BioResource Center at Cornell University (Ithaca, NY) exactly as previously described^52^. The samples were digested with the restriction enzyme *Ape*KI. DNA fragments were barcoded and sequenced using Illumina HiSeq. 2500 (Illumina, San Diego, CA). Contamination was checked using a blank sample randomly placed in the sequencing plate (one per plate); blanks should have less than 10% of the mean barcode reads value (MBRV). Sample Y20140298 has less than 10% of the MBRV and was removed from the data set.

The raw GBS reads were analysed using the default parameter of the TASSEL pipeline version 5.0^52–54^ (http://www.maizegenetics.net). Tags were aligned to the reference genome (Nipponbarre, MSU version 7.0) using the Burrows-Wheeler Aligner version 0.7.8-r455 (BWA)^55^. SNPs were identified from the Tags using the plugin TagsToSNPByAlignment and identical SNPs were merged using the MergeDuplicateSNPs plugin^54^. Imputation of missing data was performed with the FastImputationBitFixedWindow plugin using the default settings as of Begum^56^. SNPs missing in more than 25% of the data set were filtered along with SNPs with minor allele frequency (MAF) of 5%, with a resulting data matrix of 33, 725 SNPs.

#### Genome-wide Association Study

Association mapping was performed in TASSEL 5.0^54^ using the mixed-linear model (MLM) with the log_10_-transformed peak area of the target metabolites as the ‘trait’. Corrections for confounding effects due to subpopulation structure and relatedness between individuals were done by performing PCA^57^ and calculating the kinship matrix (k)^58^, using the (PCA + K) workflow in TASSEL 5.0^54^. The first five components of the PCA were used as covariates in the MLM and the kinship matrix was generated using the Centered_IBS method^59^. Manhattan plots and qq plots were also generated in TASSEL 5.0. Significance thresholds were set at SNPs with LOD scores ≥5, where there were also at least five other SNPs with LOD scores ≥4 within 200kb from the MS SNP. LD in the QTL on chromosome 1 was estimated by standardised disequilibrium coefficients (*D*’^60^). Plots were generated using TASSEL.

#### Genotyping of FGR locus

Several mutations that lead to a non-functional AADH have been identified in rice on different genetic backgrounds^2, 8, 61, 62^. In this study, we screen for the major allele the recessive *fgr* allele, conferring fragrance, which is characterised by a 7 bp deletion and three SNPs in the seventh exon of the *FGR* gene^8^. We utilised previously developed markers^29^ to genotype Set 2 collection for this allele. This data was added to the 33,275 SNP matrix and GWAS was rerun for target compounds.

### Statistical analyses

After pre-processing the metabolomics data, the mean values of the two technical replicates were used for further statistical analysis and GWA. For multivariate analysis, the data were log transformed to reduce the skewness of the variables^63^. The data was then pareto scaled to moderate the influence of compounds produced at high concentrations and recognise the effects of compounds produced at relatively lower concentrations in the overall characterisation of rice volatile compounds^64^. To obtain an overview of the data sets, the data were represented by unsupervised modelling through principal components analysis (PCA) using Simca-P. Next, to determine the discriminating compounds in the aromatic rice varieties used in this study (Set 1), orthogonal partial least squares-discriminant analysis (oPLS-DA) was conducted, again in Simca-P. OPLS-DA maximises the covariance of the matrix of predictor data, X, and the response block, Y, thus providing a more powerful prediction of among-group variation compared to PCA^65^. Compounds with the top five VIP scores were examined in greater detail. Finally, correlation analysis was performed for the top five discriminating compounds of both sample sets using the “cor” function in R^66^. To compare the relative amounts of the five amine heterocycles between the two sample groups (Set 1 n = 11; and Set 2 n = 341), a full model, accounting for sample group and compound, was fit to the data using the linear mixed effects ‘lme4’ R package^67^. In the usual fashion, a second, nested model, which omitted sample group as predictor variable, was fit, and the two models were compared.

### Data availability

Data files are accessible through the UQ espace digital repository http://dx.doi.org/10.14264/uql.2017.174.

## Electronic supplementary material

Supplementary Information

Dataset 1
