# Supplementary material for: Metabolomics and genomics combine to unravel the pathway for the presence of fragrance in rice
Source: Sci Rep. 2017 Aug 18;7:8767. doi: 10.1038/s41598-017-07693-9 (PMC5562744; doi:10.1038/s41598-017-07693-9)

Supplementary Information: Metabolomics and genomics combine to unravel the pathway for the presence of fragrance in rice.

Venea Dara Daygon, Mariafe Calingacion, Louise C. Forster, James J. De Voss, Brett D. Schwartz, Ben Ovenden, David E. Alonso, Susan R. McCouch, Mary J. Garson, Melissa A. Fitzgerald

Supplementary Information S1. Methodology of chemical synthesis.

A. 2AP

2-(Methoxycarbonyl)-1-pyrroline (**6**)

Following a method identical to that of De Kimpe (1993), To a solution of methyl prolinate **7** (1.94 g, 15.1 mmol) in anhydrous ether (130 mL) at 0 ºC, was added drop wise tert-butylhypochlorite (1.67 g, 15.4 mmol) and the reaction then stirred for 1 hour. The reaction was checked by GC-MS to monitor for the formation of the *N*-chloro product, then triethylamine (1.55 g, 15.4 mmol) was added drop wise and stirred at room temperature overnight. The mixture was filtered through a bed of celite then concentrated *in vacuo* to afford an oil which was purified by short-path Kugelrohr distillation (bp. 70 - 90 ºC, 160 mmHg [lit bp. 41-44 ºC, 13 mmHg]) to give 2-(methoxycarbonyl)-1-pyrroline (244.7 mg, 13%) as a colourless oil with spectroscopic data identical to those reported in the literature by De Kimpe (1993).

2-Acetyl-1-pyrroline (**1**) and 6-methyl-5-oxo-2,3,4,5-tetrahydropyridine (**5**)

Synthesised using a method similar to that of De Kimpe (1993): To a solution of **6** (45 mg, 3.5 mmol) in anhydrous ether (5 mL) under nitrogen at -15 ºC (salt / ice) was added methylmagnesium bromide (140 µL, 4.0 mmol, 3 M solution in ether) dropwise over 5 mins. The reaction was monitored over 2.5 hours by GC-MS, then quenched with 1 M HCl and stirred for 30 min. The ethereal layer was then removed and discarded and the aqueous layer neutralised with 2 M NaOH and basified to pH 10. The mixture was extracted with DCM (5 × 5 mL), dried over anhydrous sodium sulfate, filtered and stored in the freezer in DCM. The crude residue was examined by GC-MS and by ^{1}H NMR, revealing the presence of traces of the double addition product. A portion of the final product mixture was purified by silica flash chromatography using silica that was pre-washed with hexane/triethylamine (10 mL, 99:1). After application of the product mixture to the column, fractions were eluted with ether:pentane (1:3), combined and concentrated to give a mixture of 2AP and 6M5OTP, together with trace amounts of tertiary alcohol by-product, as a malodorous slightly pale yellow oil. This was then carefully concentrated under nitrogen, diluted in $CDCl_3$ and and subjected to acid-base partitioning by extraction into 1 M HCl, followed by basification to pH 10 using 1 M NaOH, and extraction back into deuterated chloroform. GC-MS and ^{1}H NMR data were run, showing a ratio of 76:24 (GC-MS) and 78:22 (^{1}H NMR). The remaining unpurifed sample was stored frozen in solution in DCM at -78 ºC.

2-Acetyl-1-pyrroline: ^{1}H NMR (CDCl3, 700 MHz) δ_H 1.93-1.97 (2H, m, H_2-4), 2.49 (3H, s, CH_3), 2.72-2.75 (2H, m, H_2-3) and 4.11- 4.13 (2H, tt, *J* = 15.0, 7.5, 2.5 Hz, H_2-2) ; ^{13}C NMR ($CDCl_3$, 700 MHz) δ_C 197.9, 174.5, 62.6, 33.2, 26.1, 22.2; GC-MS 111 (14), 83 (35), 69 (15), 68 (17), 55 (4)

6-Methyl-5-oxo-2,3,4,5-tetrahydropyridine: ^{1}H NMR (CDCl3, 700 MHz) δ_H 2.08-2.12 (2H, m, H_2-3), 2.12 (3H, t, *J* = 2.0 Hz, CH_3), 2.52 (2H, t, *J* = 6.3 Hz, H_2-4) and 3.84-3.86 (2H, m, H_2-2) ; ^{13}C NMR ($CDCl_3$, 700 MHz) δ_C 191.7, 164.8, 49.9, 36.6, 23.7, 20.1; GC-MS 111 (22), 83 (29), 56 (4).

B. 1-Pyrroline (**4**)

Following a method identical to that of Ottinger and Hofmann (2002): To a solution of L-proline (2.58 g, 22.4 mmol) in water (40 mL), was added to an aqueous solution of sodium metaperiodate (50 mL, 0.3 mmol/L) and the reaction then stirred for 2 hours in the dark at room temperature. The pH of the reaction was then adjusted to 9 with 1 M aqueous sodium hydroxide. The solution was extracted with diethyl ether (3 × 20 mL), and the combined organic layers then washed with brine (20 mL). After the organic layer was dried over Na_2SO_4 it was concentrated under a gentle stream of nitrogen. A portion of the product mixture was carefully concentrated under nitrogen and rapidly purified on a neutral alumina pipette column (slightly deactivated, pre-washed with *n*-pentane/water; 5 mL, 99:1). After flushing the column with 100% *n*-pentane (5 mL), 1-pyrroline was eluted with *n*-pentane:diethyl ether (8:2). GC-MS and ^{1}H NMR data were collected. 1-Pyrroline was stored frozen in solution in *n*-pentane/ether (8:2) at -20 ºC.

1-Pyrroline (4): ^{1}H NMR ($CDCl_3$, 500 MHz) δ_H 1.82 (2H, m, H_2-3), 2.54 (2H, m, H_2-2) 3.85 (2H, m, H_2-4) and 7.62 (1H, m, H-1); GC-MS 69 (43), 68 (29), 42 (47), 41 (100).

Supplementary Information S2. GC-MS, NMR and GC × GC-TOF-MS operating conditions.

Nuclear Magnetic Resonance Spectroscopy

Proton nuclear magnetic resonance (^{1}H NMR) spectra were recorded on a Bruker Avance 500 spectrometer using a 5 mm SEI probe or a Bruker Avance DRX 700 spectrometer with a 5 mm TXI Zgrad probe. Carbon-13 nuclear magnetic resonance (^{13}C NMR) spectra were recorded on a Bruker Avance DRX 700 spectrometer with a 5 mm TXI Zgrad probe. Measurements were made in deuterated chloroform ($CDCl_3$, referenced to: δ_H 7.26 ppm, δ_C 77.16 ppm). Chemical shifts (δ) were recorded in parts per million (ppm) and coupling constants (*J* values) were measured in Hertz (Hz). Two dimensional NMR (2D NMR) data were acquired from Bruker Avance 500 and 700 MHz instruments. Gradient enhanced HMBC (geHMBC) and HSQC (geHSQC) NMR were obtained with 8 to 64 transients per increment with the evolution delay set at $^{n}J_{CH}$ of 4 Hz or 8 Hz (geHMBC) and $^{1}J_{CH}$ of 135 Hz (geHSQC). Gradient COSY (gCOSY) was recorded with 8 to 32 transients per increment with a pulse delay of 2.0 seconds.

Gas Chromatography/Mass Spectrometry

Gas chromatography/mass spectrometry (GC/MS) spectra were recorded on a Shimadzu GC-MS-QP2010 Plus. GC/MS programme: flow rate 1.5 mL/min; initial oven temperature 100 ^{o}C (isothermal for 3 minutes); ramped 16 ^{o}C/min to 270 ^{o}C held for 10 min; injection temperature 250 ^{o}C.

Comprehensive Two-dimensional Gas Chromatography/Time-of-flight Mass Spectrometry

Synthesised authentic standards (1 µL) were allowed to equilibrate in 10ml GC vials for two hours without heating and agitation. The headspace (500 µL) was collected using a 2.5 mL headspace syringe and injected on a Leco Pegasus 4D GC × GC-TOF-MS (St. Joseph, MI, USA) in splitless mode. The inlet and transfer line temperatures were set at 250 and 240 ^{o}C, respectively. The primary oven temperature was held initially at 45 ^{o}C for 1 min and then ramped at a rate of 10 ^{o}C/min to 235 ^{o}C. The secondary oven and the modulator were set at 15 and 25 ^{o}C higher than the primary oven, respectively for the entire run. The modulation period was set at 2.5 s, with a hot pulse of 0.4 s and 0.85 s cold pulse between stages. Data acquisition was done in full scan at 1500 V and 70 V electron

energy. The ion source was set at 240 ^{o}C. TOF-MS autotuning (acquisition system adjustment, ion optic focusing and mass calibration) was done every 48 hours. The total analysis time is 30 mins.

Table S1. Information on samples included in Set 2, including variety name, pedigree information, FGR allele, and peak areas of the compounds.

Taxa	Pedigree	*FGR* allele	acetoin	1-pyrroline (Analye 4)	Pyrrole	2AP	6M5OTP (Analyte 5)	2-acetylpyrrole
AZUCENA	#N/A	#N/A	51553.02	9753.54	13043.26	183313.06	69345.01	66760.055
Baru	#N/A	#N/A	1	1	1	734.135	1	1
Basmati_370	#N/A	#N/A	11945.245	1	10279.16	33556.066	7023.7	3741.765
BENGAL	#N/A	#N/A	1	1	1978.765	4043.43	1418.86	2388.7
CALMATI_201	#N/A	#N/A	1	43068.625	18097.88	30452.87	7708.81	4872.24
DELLA	#N/A	#N/A	1	1	1	1400.63	1	1
DELLMONT	#N/A	#N/A	7681.975	18167.926	8097.56	92207.945	19626.795	17922.28
DELLROSE	#N/A	#N/A	7866.265	181074.5	14578.3	128181.52	50904.34	41068.414
DOONGARA	#N/A	#N/A	1	1	2058.47	7248.855	1704.035	6464.055
DULAR	#N/A	#N/A	11067.95	1	1662.115	4210.57	1	1804.275
GOOLARAH	#N/A	#N/A	1	37845.18	13568.28	166882	60648.773	59345.67
HOM_MALI_NIAW	#N/A	#N/A	15907.39	5249.935	5690.6	46780.19	11184.345	10045.08
HOM_NANG_NEAUNG	#N/A	#N/A	12055.675	14092.48	11577.31	185398.56	69819.39	11309.91
Illabong	#N/A	#N/A	1	1	1	2741.635	1	1
Jarrah	#N/A	#N/A	19464.325	1	1526.485	1993.35	1	1
JEFFERSON	#N/A	#N/A	4119.615	1	1	1	1	1
Kulu	#N/A	#N/A	21842.045	1	1	1	1	1
KYEEMA	#N/A	#N/A	1	101157.69	33433.57	91890.76	21484.42	14224.62
L_202	#N/A	#N/A	1	1	3231.91	1444.645	1	1396.14
LANGI	#N/A	#N/A	1	1	5440.56	4919.945	1914.91	1207.595
M_9	#N/A	#N/A	10881.105	1	3506.755	3262.955	1949.33	1870.465
M104	#N/A	#N/A	10891.84	1	1967.32	714.285	1	1
M205	#N/A	#N/A	1	1	1834.51	2757.02	1	3227.45
MILLIN	#N/A	#N/A	8260.535	1	2084.345	3100.94	1	2584.195
NORIN_PL_8	#N/A	#N/A	11768.57	1	4090.94	2839.64	1	1337.075
Opus_	#N/A	#N/A	6814.11	1	1	1969.29	1	1
PANDAN_WANGI_7__Y1_	#N/A	#N/A	5078.44	1	8814.555	86772.58	16981.54	44195.914

PANDAN_WANGI_8__Y3_	#N/A	#N/A	8885.84	22166.984	7529.695	36094.45	6762.165	2290.085
PANDAN_WANGI_9__Y4_	#N/A	#N/A	9227.8	7589.51	6408.145	29990.4	5989.315	1
PELDE	#N/A	#N/A	14857.38	1	1	861.92	1	1
PHKA_RUMDUOL	#N/A	#N/A	7556.502	28776.781	10383.84	204645.7	62533.61	75903.01
Quest	#N/A	#N/A	1666002.89	1	2165.4	5070.14	1	2232.13
Reiziq	#N/A	#N/A	642526.805	1	1	1	1	1
Sherpa	#N/A	#N/A	9013.305	1	1746.51	2079.32	1	2068.935
Takanari	#N/A	#N/A	13239.415	1	5101.705	2377.4	1	1
Y20140001	BENGAL/PANDAN WANGI (7)	fgr	1	256318.97	9752.59	76923.11	14194.415	13075.28
Y20140002	BENGAL/PANDAN WANGI (7)	Fgr	5158.345	11030.7	3268.21	5662.54	1523.69	5956.98
Y20140003	BENGAL/PANDAN WANGI (7)	Fgr	3816.105	1	1886.99	3424.865	1	1385.64
Y20140004	BENGAL/PANDAN WANGI (7)	#N/A	13751.96	8609.42	5215.345	7949.855	2895.825	4185.64
Y20140005	BENGAL/PANDAN WANGI (7)	Fgr	1	1	1980.8	2830.165	1	1555.79
Y20140006	BENGAL/PANDAN WANGI (7)	Fgr	1	17626.164	4088.245	4243.705	1	3555.965
Y20140007	BENGAL/PANDAN WANGI (7)	Fgr	1	6550.7	1	1739.915	1	1
Y20140009	BENGAL/PANDAN WANGI (7)	fgr	1	32792.58	9943.131	132561.64	39848.49	22403.795
Y20140010	BENGAL/PANDAN WANGI (7)	fgr	1	157929.55	10800.08	71324.32	12432.245	9965.625
Y20140011	BENGAL/PANDAN WANGI (7)	fgr	1	49854.21	11810.46	155429.73	54859.547	16900.39
Y20140012	BENGAL/PANDAN WANGI (7)	fgr	1	42482.37	12184.84	35172.89	8388.375	8377.61
Y20140013	BENGAL/PANDAN WANGI (7)	Fgr	1	1	2705.84	13434.21	1660.09	4977.105
Y20140014	BENGAL/PANDAN WANGI (7)	fgr	1	5332.885	8326.975	46318.14	8228.56	4711.265
Y20140015	BENGAL/PANDAN WANGI (7)	fgr	1	52607.516	11474.55	156571.55	47076.91	42854.465
Y20140016	BENGAL/PANDAN WANGI (7)	Fgr	4549.5	1	3026.33	122908.47	20879.27	47762.5
Y20140017	BENGAL/PANDAN WANGI (7)	fgr	12815.431	10839.656	1	4852.16	1	2647.069
Y20140018	BENGAL/PANDAN WANGI (7)	fgr	3457.515	15179.82	15644.36	267211.88	103201.95	49811.227
Y20140019	BENGAL/PANDAN WANGI (7)	fgr	4517.415	466292.8	14472.95	149524.28	41350.297	84468.21
Y20140020	BENGAL/PANDAN WANGI (7)	Fgr	1	42655.098	1	39570.664	6878.775	1
Y20140021	BENGAL/PANDAN WANGI (7)	Fgr	1	1	1	4598.415	1398.84	1677.745
Y20140022	BENGAL/PANDAN WANGI (7)	Fgr	4364.385	1	1	4732.755	1	1901.505
Y20140023	BENGAL/PANDAN WANGI (7)	Fgr	8798.53	26218.94	6373.985	5908.27	1656.45	3119.61

Y20140024	BENGAL/PANDAN WANGI (7)	fgr	1	16424.346	6886.795	64287.27	9665.53	12742.82
Y20140025	BENGAL/PANDAN WANGI (7)	fgr	1	30367.71	11351.12	90345.664	16283.835	17839.535
Y20140026	BENGAL/PANDAN WANGI (7)	#N/A	19657.025	24163.346	6701.465	2921.92	1	1
Y20140027	BENGAL/PANDAN WANGI (7)	#N/A	21325.095	1	4160.87	6803.805	1793.8	1
Y20140028	DOONGARA/PANDAN WANGI (7)	fgr	1	1	13362.06	204690.94	82033.27	71251.47
Y20140030	DOONGARA/PANDAN WANGI (7)	Fgr	1	5115.345	2148.12	16266.655	2085.74	6207.68
Y20140031	DOONGARA/PANDAN WANGI (7)	Fgr	1	1	9232.838	228051.47	60121.688	81864.836
Y20140033	DOONGARA/PANDAN WANGI (7)	Fgr	9491.285	1	3971.445	3183.515	1	1306.545
Y20140034	DOONGARA/PANDAN WANGI (7)	Fgr	1	1	1	2384.065	1	1169.085
Y20140035	DOONGARA/PANDAN WANGI (7)	Fgr	1	1	1	1783.33	1	1214.84
Y20140036	DOONGARA/PANDAN WANGI (7)	Fgr	1	1	4123.645	5468.595	1497.215	2320.82
Y20140037	DOONGARA/PANDAN WANGI (7)	fgr	1	25151.47	10992.05	45952.06	9189.23	6026.985
Y20140038	DOONGARA/PANDAN WANGI (7)	Fgr	10404.87	1	4823.76	1	1	1
Y20140039	DOONGARA/PANDAN WANGI (7)	fgr	9382.675	1	14028.46	113193.805	18716.244	7080.455
Y20140040	DOONGARA/PANDAN WANGI (7)	fgr	1	12300.3	9918.185	88545.89	16235.715	8261.365
Y20140041	JEFFERSON/AZUCENA	Fgr	1	1	1	1860.66	1	1
Y20140042	JEFFERSON/AZUCENA	Fgr	1	1	4034.054	4428.24	1	1
Y20140043	JEFFERSON/AZUCENA	Fgr	9629.71	1	3334.275	3925.755	1	1327.205
Y20140044	JEFFERSON/AZUCENA	fgr	1	37539.09	12509.81	147045.97	46455.555	52956.656
Y20140045	JEFFERSON/AZUCENA	Fgr	1	1	2252.895	2229.03	1	1
Y20140046	JEFFERSON/AZUCENA	#N/A	4241.285	441127.12	22270.15	232879.27	79273.92	77628.766
Y20140047	JEFFERSON/AZUCENA	Fgr	1	1	1712.57	1223.08	1	1
Y20140048	JEFFERSON/AZUCENA	Fgr	1	1	1	1801.025	1	1181.32
Y20140049	JEFFERSON/AZUCENA	fgr	4863.205	198400.33	70014.43	332540.56	132742.4	143287.72
Y20140050	JEFFERSON/AZUCENA	fgr	8707.125	29336.49	18468.4	248309.34	96247.56	97786.305
Y20140051	JEFFERSON/AZUCENA	Fgr	1	1	1677.085	6112.355	1804.405	3989.575
Y20140052	JEFFERSON/AZUCENA	Fgr	3276.625	1	2951.32	4389.875	1793.92	4070.755
Y20140053	JEFFERSON/AZUCENA	fgr	1	86178.33	19808.74	225692.34	83904.32	74914.75
Y20140054	JEFFERSON/AZUCENA	fgr	1	58018.47	17344.89	210745.94	67648.89	90569.37
Y20140055	JEFFERSON/AZUCENA	fgr	2932.88	51013.19	17741.82	275875.34	104421.88	109178.58
Y20140056	JEFFERSON/AZUCENA	fgr	11528.78	192137.8	14665.01	111478.875	19867.885	9928.13
Y20140057	JEFFERSON/AZUCENA	fgr	1	166021.55	14632.56	54520.066	11165.76	10115.14
Y20140058	JEFFERSON/AZUCENA	fgr	1	45339.56	14951.1	43640.945	8556.04	4996.675

Y20140059	JEFFERSON/AZUCENA	Fgr	1	1	1	1827.68	1	1
Y20140060	JEFFERSON/AZUCENA	Fgr	1	1	7715.67	46155.31	10393.68	8514.73
Y20140061	JEFFERSON/AZUCENA	fgr	8993.302	20273.4	21628.44	267563.03	106636.85	81880.99
Y20140062	JEFFERSON/AZUCENA	fgr	1	21245.635	16127.7	202669.56	66619.336	68509.32
Y20140063	JEFFERSON/AZUCENA	fgr	11056.94	268654.94	46963.28	754059.9	260460.02	281586.34
Y20140064	JEFFERSON/AZUCENA	Fgr	1	1	5477.92	5471.435	2160.435	1946.12
Y20140065	JEFFERSON/AZUCENA	fgr	10890.91	10097.2	11043.63	244487.67	74905.6	86728.29
Y20140066	JEFFERSON/AZUCENA	fgr	6658.485	209429.84	14098.44	243195.16	78495.4	97921.57
Y20140067	JEFFERSON/AZUCENA	Fgr	1	1	6288.27	6697.23	1631.215	4402.565
Y20140068	JEFFERSON/AZUCENA	Fgr	9787.75	1	2344.31	3052.91	1	1
Y20140069	JEFFERSON/AZUCENA	fgr	1	48776.19	18913.27	23610.94	6480.91	5517.08
Y20140070	JEFFERSON/AZUCENA	fgr	1	258654.75	14291.13	64614.297	13080.775	10485.54
Y20140071	JEFFERSON/AZUCENA	fgr	3195.85	46836.22	66671.81	261935.67	103647.08	81910.28
Y20140072	JEFFERSON/AZUCENA	Fgr	8705.575	419732.16	17537.76	289562.28	92274.28	105070.76
Y20140073	JEFFERSON/AZUCENA	Fgr	10433.38	5051.555	1643.25	3480.67	1	2925.455
Y20140074	JEFFERSON/AZUCENA	Fgr	1	1	1	1	1	1
Y20140075	JEFFERSON/AZUCENA	Fgr	10849.13	1	4042.89	1172.76	1	1
Y20140076	JEFFERSON/AZUCENA	fgr	1	547513.44	18901.15	140851.9	33919.477	36835.125
Y20140077	JEFFERSON/AZUCENA	Fgr	1	1	6326.545	5522.93	1823.94	1748.7
Y20140078	KYEEMA/BASMATI 370	fgr	11227.465	365396	18550.6	293553.75	96295.47	117380.84
Y20140079	KYEEMA/BASMATI 370	fgr	1	36292.945	18348.94	31934.38	5994.885	1382.005
Y20140080	KYEEMA/BASMATI 370	fgr	9550.585	462633.72	15806.98	134005.61	33198.836	38527.305
Y20140081	KYEEMA/BASMATI 370	fgr	3858.82	292163.38	13653.42	177849.14	54235.754	67444.58
Y20140082	KYEEMA/BASMATI 370	fgr	3875.59	174196.31	12129.05	144298.2	41159.113	52856.96
Y20140083	KYEEMA/BASMATI 370	fgr	1	38317.47	13973.62	49890.406	11221.3	10147.045
Y20140084	KYEEMA/BASMATI 370	fgr	31702.35	324287.56	18434.22	176361.47	65210.51	62614.844
Y20140085	KYEEMA/BASMATI 370	fgr	1	118625.31	18822.12	79065.54	14558.55	10007.67
Y20140087	KYEEMA/BASMATI 370	fgr	1	1	3723.3	56183.023	11525.475	9799.535
Y20140088	KYEEMA/BASMATI 370	fgr	6749.53	811706.56	19438.1	685848.4	197339.61	230966.75
Y20140089	KYEEMA/BASMATI 370	fgr	1	246477.14	16549.05	105331.66	28614.18	14573.63
Y20140090	KYEEMA/BASMATI 370	fgr	12571.95	700984.06	20953.59	168819.38	56894.15	58386.4
Y20140091	KYEEMA/BASMATI 370	fgr	1	36377.3	20537.87	138290.23	42746.1	51676.867
Y20140092	KYEEMA/BASMATI 370	fgr	3251.275	661867.3	45077.66	183185.23	68207.85	53457.613

Y20140093	KYEEMA/BASMATI 370	fgr	1	24262.375	9073.769	44773.906	8874.1	9062.774
Y20140094	KYEEMA/BASMATI 370	fgr	1	50871.12	17541.93	95784.42	19937.436	10142.49
Y20140095	KYEEMA/BASMATI 370	fgr	9143.315	233817.1	52061.35	299520.47	105959.56	111912.42
Y20140096	KYEEMA/BASMATI 370	fgr	3040.51	31848.75	9318.745	177942.8	56995.49	66506.12
Y20140097	KYEEMA/BASMATI 370	fgr	8843.21	174729.19	13985.61	89978.63	20255.83	37637.016
Y20140098	KYEEMA/BASMATI 370	fgr	9458.83	623688.6	20995.85	111301.08	24460.83	30444.006
Y20140099	KYEEMA/BASMATI 370	fgr	9689.905	151074.19	16349.27	177248	52937.98	68480.92
Y20140100	KYEEMA/BASMATI 370	fgr	1	230693.19	12461.86	88511.44	19596.23	13021.89
Y20140101	KYEEMA/BASMATI 370	fgr	1	28463.24	8111.3	48857.555	10651.545	7280.475
Y20140102	KYEEMA/BASMATI 370	fgr	30410.495	48248.934	8535.475	199361.03	60403.28	82444.53
Y20140103	KYEEMA/BASMATI 370	fgr	1	461525.78	19496.43	59311.02	14033.205	8102.25
Y20140104	KYEEMA/BASMATI 370	fgr	6491.431	441934.03	66051.75	208427.58	73989.96	73108.28
Y20140105	KYEEMA/BASMATI 370	fgr	8268.39	65287.227	24021.21	101944.625	16964.91	6398.75
Y20140106	KYEEMA/BASMATI 370	fgr	1	1	10001.11	65689.51	13094.03	10548.67
Y20140107	KYEEMA/BASMATI 370	fgr	1	478883.1	22783.52	158264.97	34589.684	29320.78
Y20140108	KYEEMA/BASMATI 370	fgr	1	127376.4	20871.56	160933.1	58319.344	67387.195
Y20140109	KYEEMA/BASMATI 370	fgr	10002.53	585834.6	61051.33	154724.72	57693.45	17367.855
Y20140110	KYEEMA/BASMATI 370	fgr	8097.144	514221.4	59943.29	325693.56	128154.03	114635.73
Y20140111	KYEEMA/BASMATI 370	fgr	4320.86	190032.61	26302.81	246454.27	104775.76	106017.625
Y20140112	KYEEMA/BASMATI 370	fgr	1	23761.705	5831.88	193038.44	64426.38	72844.87
Y20140117	KYEEMA/KDML 105	fgr	1	26096.23	11476.47	161156.56	51217.785	58126.746
Y20140118	KYEEMA/KDML 105	fgr	18591.105	719819.94	48386	662168.5	217139.69	266148.84
Y20140119	KYEEMA/KDML 105	fgr	7485.79	155454.11	21042.65	239611.55	86572.75	89936.57
Y20140120	KYEEMA/KDML 105	fgr	4026.655	119584.23	20328.09	207504.69	79542.39	47827.9
Y20140121	KYEEMA/KDML 105	fgr	1	32877.305	17945.37	93092.1	21242.285	16341.405
Y20140122	KYEEMA/KDML 105	Fgr	1	53044.066	21779.03	201468.75	76671.48	77748.63
Y20140123	KYEEMA/KDML 105	fgr	7024.085	10951.58	8288.435	116025.49	22393.58	63986.695
Y20140124	KYEEMA/KDML 105	fgr	1	198558.38	16842.18	103968	21269.5	17499.61
Y20140125	KYEEMA/KDML 105	Fgr	1	1	3163.975	2247.275	1	1
Y20140126	KYEEMA/KDML 105	fgr	4238.98	128710.59	17089.21	231478.94	86543.48	92206.98
Y20140127	KYEEMA/KDML 105	fgr	1	15006.385	4940.2	66732.12	12525.76	13391.23
Y20140129	KYEEMA/KDML 105	#N/A	13560.323	12041.003	11684.55	551619.4	167639.17	197425.78
Y20140130	KYEEMA/KDML 105	fgr	3398.06	33012.266	17884.78	397783.3	130334.29	150572.06

Y20140131	KYEEMA/KDML 105	fgr	10236.78	187478.6	13520.3	289992	94468.79	102957.625
Y20140132	KYEEMA/KDML 105	fgr	11548.578	918473.9	22299.03	360634.72	141771.4	145683.33
Y20140133	KYEEMA/KDML 105	fgr	8752.73	31331.955	11945.77	160871.14	53150.184	56983.477
Y20140134	KYEEMA/KDML 105	fgr	1	37298.27	19036.46	107986.17	21305.74	15638.15
Y20140135	KYEEMA/KDML 105	fgr	1	48306.33	14402.4	64748.996	12488.82	7300.53
Y20140136	KYEEMA/KDML 105	fgr	8734.315	92319.2	20483.23	264863.25	93199.59	97806.44
Y20140137	KYEEMA/KDML 105	fgr	8864.079	66222.23	23682.35	197461.66	75659.58	72325.49
Y20140138	LANGI/BASMATI 370	fgr	19141.035	382671.4	48317.81	339721.56	134159.44	128116.83
Y20140139	LANGI/BASMATI 370	fgr	16905.645	30154.936	18151.19	319610.6	128175.53	71452.8
Y20140140	LANGI/BASMATI 370	fgr	9446.445	460863.28	39611.66	243307.83	81496.27	97122.086
Y20140141	LANGI/BASMATI 370	Fgr	1	1	4742.59	3000.755	1	2489.38
Y20140142	LANGI/BASMATI 370	fgr	10503.06	29244.65	12666.19	61054.184	12160.16	8764.425
Y20140143	LANGI/BASMATI 370	Fgr	8403.655	1	5424.75	1993.265	1	1
Y20140145	LANGI/BASMATI 370	Fgr	1	31576.404	17029.92	72735.625	14692.83	13980.515
Y20140146	LANGI/BASMATI 370	Fgr	1	1	7005.275	6371.11	2434.54	4121.045
Y20140147	LANGI/BASMATI 370	Fgr	8963.595	21327.89	6013.8	6337.35	1616.73	4570.765
Y20140148	LANGI/BASMATI 370	Fgr	3614.545	1	2710.26	2866.53	1	1
Y20140150	LANGI/BASMATI 370	fgr	9803.98	414123.97	38125.46	139369.66	53595.23	64201.844
Y20140152	LANGI/BASMATI 370	fgr	1	1	7338.23	5794.33	3144.97	2605.01
Y20140153	LANGI/BASMATI 370	Fgr	4007.86	1	2530.53	4300.905	1694.76	2858.02
Y20140154	LANGI/BASMATI 370	fgr	1	32708.639	13553.94	73302.92	11194.747	8048.032
Y20140155	LANGI/BASMATI 370	fgr	17030.835	16623.795	7229.21	169201.1	53190.26	60411.805
Y20140156	LANGI/BASMATI 370	#N/A	23598.56	110246.77	19193.43	118008.09	22387.5	28784.404
Y20140157	LANGI/BASMATI 370	fgr	9153.03	244382.14	21041.38	221401.98	85449.95	71198.945
Y20140158	LANGI/BASMATI 370	fgr	4205.925	25671.586	16121.71	182420.31	64716.98	75216.63
Y20140159	LANGI/BASMATI 370	Fgr	1	1	1	1	1	1
Y20140160	LANGI/BASMATI 370	Fgr	7175.414	16719.686	1	3029.953	1	1
Y20140161	LANGI/BASMATI 370	fgr	3552.72	30571.215	12100.59	124388.2	22964.436	29096.645
Y20140162	LANGI/BASMATI 370	fgr	1	33450.434	10944.85	68335.56	15602.725	11699.42
Y20140165	LANGI/BASMATI 370	fgr	4926.01	35359.48	12540.99	185419.88	70465.414	69808.04
Y20140166	LANGI/BASMATI 370	Fgr	8118.27	521731.38	39666.61	266744.6	88057.99	105579.76
Y20140167	LANGI/BASMATI 370	fgr	4507.87	453103.06	15940.49	125757.59	24292.47	54024.195
Y20140169	LANGI/BASMATI 370	fgr	9260.515	373764.88	17145.91	87078.45	21008.64	15033.41

Y20140170	LANGI/BASMATI 370	fgr	1	32317.047	12853.3	67046.41	14534.848	9040.019
Y20140171	LANGI/BASMATI 370	fgr	17369.7	480682.9	52800.84	101756.75	17876.775	16537.705
Y20140172	LANGI/BASMATI 370	fgr	1	42851.305	19436.38	139864.89	52112.01	11223.975
Y20140173	LANGI/BASMATI 370	fgr	11045.115	232048.62	27698.02	258424.81	97817.3	91758.62
Y20140174	LANGI/BASMATI 370	fgr	13655.815	924820.44	22265.65	55403.246	12698.285	11987.39
Y20140175	LANGI/BASMATI 370	Fgr	3727.075	1	2209.65	2495.28	1	1
Y20140176	LANGI/BASMATI 370	fgr	3827.735	44601.88	12120.29	112231.98	19278.49	28886.15
Y20140177	LANGI/BASMATI 370	fgr	1	44622.65	23606.9	223487.55	93512.664	55818.637
Y20140178	LANGI/BASMATI 370	fgr	3343.135	241771.31	22768.21	97373.41	16202.92	9684.395
Y20140179	LANGI/BASMATI 370	fgr	9551.06	154079.31	12177.39	38996.273	8475.504	7398.891
Y20140180	LANGI/BASMATI 370	Fgr	12766.895	23160.03	5078.075	1974.97	1	1
Y20140181	LANGI/BASMATI 370	Fgr	1	1	1	2394.195	1	5688.603
Y20140182	LANGI/BASMATI 370	Fgr	13537.975	1	6500.445	4118.2	1	5014.39
Y20140183	LANGI/BASMATI 370	Fgr	7408.97	1	1	1671.03	1	2102.67
Y20140184	LANGI/BASMATI 370	Fgr	11170.235	22267.76	8395.96	3850.07	1	2752.885
Y20140185	LANGI/BASMATI 370	Fgr	1	12831.33	3743.395	6295.08	1760.195	4846.02
Y20140186	LANGI/BASMATI 370	fgr	1	49982.766	17720.97	98940.65	19540.33	15810.01
Y20140187	LANGI/BASMATI 370	fgr	1	25570.365	9300.28	120403.055	21330.096	30404.22
Y20140189	LANGI/BASMATI 370	Fgr	1	1	2275.135	1480.26	1	1
Y20140190	LANGI/BASMATI 370	Fgr	4590.57	1	2166.655	3188.155	1	1646.73
Y20140191	LANGI/BASMATI 370	Fgr	1	7397.14	1575.58	784.14	1	1
Y20140192	LANGI/BASMATI 370	Fgr	34480.405	8599.05	1	4137.35	1	7091.21
Y20140193	LANGI/BASMATI 370	Fgr	16709.655	1	4577.87	915.665	1	1
Y20140195	LANGI/BASMATI 370	Fgr	1	1	1794.725	6387.095	3518.195	1804.005
Y20140196	LANGI/BASMATI 370	Fgr	1	1	2955.915	3187.46	1	3143.345
Y20140198	LANGI/BASMATI 370	Fgr	1	27578.309	6437.471	3685.167	1	3559.078
Y20140199	LANGI/BASMATI 370	Fgr	1	1	3132.335	15214.21	2455.7	1901.02
Y20140201	LANGI/BASMATI 370	Fgr	1	1	1	1738.92	1	1
Y20140202	LANGI/BASMATI 370	Fgr	1	1	2169.67	2638.03	1	1
Y20140203	LANGI/BASMATI 370	Fgr	1	1	2974.61	722.18	1	1
Y20140204	LANGI/BASMATI 370	fgr	13860.885	218045.08	15950.74	344490.88	98783.55	174608.97
Y20140205	LANGI/BASMATI 370	fgr	14612.565	303984.16	19290.21	437838.4	127842.01	147162.19
Y20140206	LANGI/BASMATI 370	fgr	1	54882.773	39147.9	73413.17	17417.836	11705.68

Y20140207	LANGI/BASMATI 370	fgr	9991.597	34445.027	18569.65	273169.56	84417.1	96735.586
Y20140208	LANGI/BASMATI 370	Fgr	215656.39	1	4339.065	6628.345	1	7322.06
Y20140209	LANGI/BASMATI 370	fgr	1	48824.83	15774.24	69044.41	15727.5	12254.77
Y20140210	LANGI/BASMATI 370	Fgr	1	1	2404.905	2952.845	1	1628.39
Y20140214	LANGI/BASMATI 370	#N/A	20972.295	10520.85	7583.76	5964.015	1	1934.97
Y20140215	LANGI/BASMATI 370	Fgr	1	1	2049.15	4925.83	1872.33	1775.215
Y20140216	LANGI/BASMATI 370	Fgr	1	1	1	757.75	1	1
Y20140217	LANGI/BASMATI 370	Fgr	11217.75	1	5013.665	1098.54	1	1
Y20140218	LANGI/BASMATI 370	Fgr	1	1	1	1016.135	1	1
Y20140219	LANGI/BASMATI 370	fgr	1	36031.395	8118.21	65508.906	15030.34	8944.16
Y20140221	LANGI/KDML 105	Fgr	1	1	3507.15	4621.365	1	3976.36
Y20140222	LANGI/KDML 105	Fgr	1	1	7466.11	8776.19	3748.6	3488.82
Y20140223	LANGI/KDML 105	fgr	9532.14	279951.78	18178.38	138128.9	44238.336	35083.36
Y20140224	LANGI/KDML 105	Fgr	1	1	4366.725	2599.095	1	1
Y20140225	LANGI/KDML 105	fgr	11217.46	35599.24	14669.47	144318.95	60374.23	7822.355
Y20140226	LANGI/KDML 105	Fgr	3643.415	1	1	892.92	1	1
Y20140227	LANGI/KDML 105	fgr	1	20946.88	9552.605	78278.77	15557.59	12274.395
Y20140228	LANGI/KDML 105	fgr	51630.804	28587.443	21670	258374.97	98045.94	94271.66
Y20140229	LANGI/KDML 105	fgr	10164.145	491926.5	21585.68	393862.8	135518.9	142545.12
Y20140231	LANGI/KDML 105	fgr	8992.945	33515.047	13791.03	168364.5	64009	68002.445
Y20140232	LANGI/KDML 105	Fgr	7976.35	1	3395.445	4222.66	1	3855.815
Y20140233	LANGI/KDML 105	fgr	12073.675	62653.63	19555.52	274810.4	98508.47	98232.53
Y20140234	LANGI/PANDAN WANGI (7)	Fgr	4131.925	1	5976.78	5568.49	1967.9	4882.255
Y20140235	LANGI/PANDAN WANGI (7)	Fgr	42787.49	33212.92	12087	877.46	1	1
Y20140237	LANGI/PANDAN WANGI (7)	#N/A	769301.02	1	5600.04	5755.985	1860.16	3068.55
Y20140238	LANGI/PANDAN WANGI (7)	Fgr	8495.695	1	3370.335	4478.87	1434.895	2466.735
Y20140239	LANGI/PANDAN WANGI (7)	fgr	7572.81	16004.015	6081.635	46151.055	9775.695	12171.575
Y20140240	LANGI/PANDAN WANGI (7)	fgr	4079.715	42795.344	22060.46	224618.38	80928.66	87666.28
Y20140241	LANGI/PANDAN WANGI (7)	Fgr	3394.3	1	1991.46	3495.78	1	1362.695
Y20140242	LANGI/PANDAN WANGI (7)	Fgr	4099.85	5004.195	4359.98	1535.73	1	1
Y20140243	LANGI/PANDAN WANGI (7)	fgr	17565.965	28908.57	12773.54	280348.06	89693.34	110471.79
Y20140244	LANGI/PANDAN WANGI (7)	Fgr	36715.435	19684.275	4398.955	2646.785	1	2060.58
Y20140245	LANGI/PANDAN WANGI (7)	fgr	8904.655	364992.88	18133.71	399433.3	139297	148471.47

Y20140246	LANGI/PANDAN WANGI (7)	fgr	6149.614	24409.695	5487.659	65307.383	13499.863	13293.333
Y20140247	LANGI/PANDAN WANGI (7)	fgr	9728.59	1	7673.695	5892.4	1353.885	4843.105
Y20140249	LANGI/PANDAN WANGI (7)	fgr	12571.987	1	9843.058	319564.4	96198.76	111118.91
Y20140251	YRL126/KDML 105	fgr	12833.235	445450.97	21153.8	311308.7	108990.96	121734.086
Y20140252	YRL126/KDML 105	Fgr	1	1	1	4873.615	1	4224.355
Y20140253	YRL126/KDML 105	Fgr	1	1	2483.355	1900.655	1	1028.575
Y20140254	YRL126/KDML 105	Fgr	13836.215	6878.81	5408.305	1891.945	1	1080.67
Y20140255	YRL126/KDML 105	Fgr	4697.355	1	4003.195	5336.265	1	9164.125
Y20140256	YRL126/KDML 105	Fgr	2729.44	1	4194.6	35609.58	7373.92	5082.12
Y20140257	YRL126/KDML 105	fgr	6842.13	255578.92	16064.27	74890.586	16149.675	15905.165
Y20140262	Pelde/Gopalbhog(4)/YR71048-10//YRL101	fgr	1	47207.77	8163.13	27322.57	6280.585	5332.31
Y20140269	PELDE/G'BHOG(4)/D.10//YRL101///YRL117/YRF203	Fgr	1	198567.39	15986.71	42738.504	10019.625	9038.07
Y20140270	PELDE/G'BHOG(4)/D.10//YRL101///YRL117/YRF203	fgr	6915.38	184022.7	12286.24	103771.6	22994.895	28250.215
Y20140271	YRF205/LANGI	fgr	8585	219303.8	19659.44	89819.9	18388.795	14279.09
Y20140272	76054/L202//DELLMONT	fgr	1	112320.01	9165.43	47497.664	9502.805	10436.735
Y20140273	76054/L202//DELLMONT	fgr	1	39824.812	12435.81	45114.754	11727.861	7870.507
Y20140274	KYEEMA///PELDE/G'BHOG(4)/D.10//YRL101	fgr	1	20995.27	10973.51	71714.98	14967.827	9626.479
Y20140275	KYEEMA///PELDE/G'BHOG(4)/D.10//YRL101	fgr	10314.635	316312	17054.76	54969.99	13166.255	8554.395
Y20140276	KYEEMA///PELDE/G'BHOG(4)/D.10//YRL101	fgr	1	31413.26	16958.01	78049.195	16223.585	10490.7
Y20140277	KYEEMA///PELDE/G'BHOG(4)/D.10//YRL101	fgr	1	180127.52	10056.21	74756.695	16355.029	14906.579
Y20140278	KYEEMA///PELDE/G'BHOG(4)/D.10//YRL101	fgr	1	31546.314	7254.535	47849.71	10109.28	9510.535
Y20140279	PELDE/G'BHOG(4)/D.10//YRL101///YRL117/YRF203	fgr	7705.815	407164.25	22101.54	109428.63	34470.57	33862.566
Y20140280	YRF208/DELLROSE	Fgr	4433.92	5153.64	1928.84	1	1	1

Y20140281	KYEEM///YRD91V55//P/GO(4)/D.10/4/CALMATI201	fgr	1	325077.03	14835.51	47084.33	8079.125	9196.14
Y20140282	YRF209/3/L202//PELDE*4/M9	Fgr	1	226384.83	15367.71	79795.8	18416.984	15503.965
Y20140283	YRF208/YRL125_CT18	fgr	9612.261	940661.06	63870.31	219466.95	82112.79	87607.65
Y20140284	YRF208/YRL125_CT18	fgr	4397.455	103127.555	14780.02	78119.8	17456.35	13943.65
Y20140285	BASMATI370/PELDE//BASMATI370///L202	Fgr	17601.435	4945.2	1	609.545	1	1
Y20140286	BASMATI370/PELDE//BASMATI370///L202	Fgr	10901.405	1	3056.855	2707.6	1	3009.72
Y20140287	L202///BASMATI 370/PELDE//BASMATI 370	fgr	1	1	1614.2	1456.14	1	1
Y20140288	L202///BASMATI 370/PELDE//BASMATI 370	Fgr	1	1	1	588.655	1	1
Y20140289	PELDE/G'BHOG(4)/D.10//YRL101///YRL117/YRF203	fgr	1	645187.4	43565.09	94355.62	18400.324	12321.98
Y20140290	PELDE/G'BHOG(4)/D.10//YRL101///YRL117/YRF203	fgr	1	467169.8	19375.88	74923.19	12754.35	12598.04
Y20140291	PELDE/G'BHOG(4)/D.10//YRL101///YRL117/YRF203	fgr	1	670328.06	24009.35	108266.91	22585.4	6679.835
Y20140292	PELDE/G'BHOG(4)/D.10//YRL101///YRL117/YRF203	fgr	1	692836.7	17505.01	53526.797	10408.085	13759.505
Y20140293	YRF208/3/DELLMONT //BASMATI 370//PELDE	fgr	4111.89	22682.02	14011.4	66704.35	13524.97	11155.185
Y20140294	YRF208//MILLIN/LIJIANGHEIGU	fgr	1	568251.5	39709.81	113953.266	22598.02	9891.415
Y20140295	YRF208//MILLIN/LIJIANGHEIGU	Fgr	1	1	1713.38	974.595	1	1
Y20140296	YRF208//MILLIN/LIJIANGHEIGU	Fgr	1	1	1	1	1	1
Y20140297	YRF208//MILLIN/LIJIANGHEIGU	Fgr	1	1	2727.91	2397.47	1	1
Y20140298	2*YRF208//MILLIN/LIJIANGHEIGU	#N/A	1	1	3344.695	3763.335	1	2788.915
Y20140299	KYEEMA/LIJIANGHEIGU	Fgr	1	209796.95	11438.02	97368.516	27679.945	16448.115
Y20140300	KYEEMA/LIJIANGHEIGU	fgr	1	45259.37	15405.99	73121.16	14693.61	9200.71
Y20140301	YRF208//MILLIN/LIJIANGHEIGU	Fgr	1	1	2041.25	2838.835	1	1230.04
Y20140302	YRF208//MILLIN/LIJIANGHEIGU	Fgr	1	1	1	910.7	1	1
Y20140303	YRF208//MILLIN/LIJIANGHEIGU	Fgr	1	1	1	1	1	1

Y20140304	YRF208//MILLIN/LIJIANGHEIGU	Fgr	1	1	1910.82	3019.935	1647.515	2839.81
Y20140305	YRF208//MILLIN/LIJIANGHEIGU	fgr	3709.345	344128.22	15629	77473.17	12675.71	28735.785
Y20140306	YRF208//MILLIN/LIJIANGHEIGU	Fgr	1	1	1	1	1	1
Y20140307	YRF208//MILLIN/LIJIANGHEIGU	Fgr	1	1	1615.505	918.475	1	1
Y20140308	YRF208//MILLIN/LIJIANGHEIGU	Fgr	1	1	3240.245	4039.16	1	4757.08
Y20140309	2*YRF208//MILLIN/LIJIANGHEIGU	fgr	19380.605	30370.69	12356.78	99613.66	19516.846	33646.51
Y20140310	2*YRF208//MILLIN/LIJIANGHEIGU	fgr	1	176267.4	14696.47	78251.22	15886.31	9561.64
Y20140311	2*YRF208//MILLIN/LIJIANGHEIGU	Fgr	1	1	1	1014.245	1	1
Y20140312	2*YRF208//MILLIN/LIJIANGHEIGU	Fgr	4583.94	1	2152.16	4919.68	1	1390.015
Y20140313	2*YRF208//MILLIN/LIJIANGHEIGU	fgr	3466.785	168559.03	13392.66	166954.11	48634.75	51232.414
Y20140314	2*YRF208//MILLIN/LIJIANGHEIGU	fgr	9992.685	276742.4	21000.16	171424.92	66844.81	15353.67
Y20140315	2*YRF208//MILLIN/LIJIANGHEIGU	Fgr	7495.405	1	1420.35	2918.115	1	1474.96
Y20140316	2*YRF208//MILLIN/LIJIANGHEIGU	Fgr	1	1	1	8082.4	3926.68	5140.435
Y20140317	2*YRF208//MILLIN/LIJIANGHEIGU	fgr	1	40821.02	10001.94	64318.19	14196.715	8971.21
Y20140318	2*YRF208//MILLIN/LIJIANGHEIGU	fgr	9610.745	632734.9	18288.7	79701.51	17388.37	9263.8
Y20140319	KYEEMA/LIJIANGHEIGU	Fgr	1	1	1513.855	3206.4	1	1272.055
Y20140320	KYEEMA/LIJIANGHEIGU	fgr	10475.28	47703.785	11552.71	53332.297	10886.94	9309.34
Y20140321	KYEEMA/LIJIANGHEIGU	#N/A	2557.982	1	4608.879	2545.902	1	1064.769
Y20140322	KYEEMA/LIJIANGHEIGU	fgr	1	15684.885	10315.59	70076.02	15281.575	11787.375
Y20140323	KYEEMA/NORIN PL8	Fgr	154902.99	1	4259.75	6033.89	1720.425	2767.41
Y20140324	KYEEMA/NORIN PL8	Fgr	1	1	2589.68	2152.54	1508.205	2419.815
Y20140325	KYEEMA/NORIN PL8	Fgr	1	1	1	1263.91	1	1619.415
Y20140326	KYEEMA/NORIN PL8	Fgr	171104.055	1	1853.92	2296.205	1	1767.445
Y20140327	KYEEMA/NORIN PL8	Fgr	31500.165	1	2232.52	4137.985	1	2543.76
Y20140360	YRK4/KOSHIHIKARI (TYNAN)//JYUODEKI	#N/A	1	1	2037.57	2322.89	1	1
Y20140361	YRK4/KOSHIHIKARI (TYNAN)//JYUODEKI	#N/A	1	1	1517.59	1794.515	1	1379.675
Y20140362	M104//YRM49/KIRARA 397	#N/A	1	1	1	2735.305	1	2363.56
Y20140363	M104//AKIHIKARI/HSC55	#N/A	3878.57	1	1	1	1	1
Y20140364	CALMOCHI101/DOONGARA//DOONGARA	#N/A	1	1	2296.83	5572.38	2072.33	2398.985

Y20140365	Doongara/Phka Rumdang/4/PELDE/G'BHOG(4)/D.10//YRL101///YRL117/YRF203	#N/A	9039.25	1	1710.755	9023.4	1816.04	7066.355
YRF_208	#N/A	#N/A	1	320803.5	60733.68	65247.18	15653.555	6134.74
YRF_209__smooth_	#N/A	#N/A	1	456586.6	23808.98	78337.74	19526.244	13647.935
YRF203	#N/A	#N/A	1	103909.45	10659.14	57663.355	11345.73	21747.494
YRF205	#N/A	#N/A	1	32823.72	15729.35	37733.586	9311.19	6730.295
YRF206	#N/A	#N/A	1	461174.97	14249.53	25130.15	5330.98	5284.225
YRL_101	#N/A	#N/A	18155.438	1	4206.598	1	1	1
YRL_117	#N/A	#N/A	1	1	2951.755	2359.315	1	1389.395
YRL_125	#N/A	#N/A	11313.785	1	1	1646.885	1	1
YRL_126	#N/A	#N/A	1	8807.885	2441.525	3843.5	1	4792.27

Table S2. GC × GC-TOF-MS parameters for comprehensive profiling of rice volatile metabolites.

**Autosampler settings**	
Incubator and agitator	On
Incubator temperature	80 °C
Fill speed	50 µl/s
Injection speed	100 µl/s
Injection volume	1.5 ml
Syringe temperature	80 °C
**GC × GC-TOF-MS conditions**	
Injection mode	Splitless
Injector temperature	250 °C
Carrier gas	Helium (99.9999%)
Flow rate	1 ml/min
Primary column	Agilent DB-624UI (midpolar)
Primary column composition	6% cyanonpropyl phenyl, 94% polydimethyl siloxane
Primary column length	30 m × 250 µ × 1.4 µ
Secondary column	Restek Stabilwax (polar)
Secondary column composition	crossbond polyethylene glycol
Secondary column length	0.9 m × 250 µ × 0.50 µ
Secondary column temperature	15 °C offset primary
Modulator temperature	25 °C offset primary
Modulation	2.5 s
Hot pulse time	0.4 s
Cool time	0.85 s
Transfer line	240 °C
MS voltage	1500 V
Electron energy	70 V
Scan rate	200 spectra/s
Mass range	35-500 *m/z*
Ion source	240 °C
**Metadata pre-processing parameters**	
Baseline offset	0.5
Peak Width	15
Match Required to combine (2D)	600
Peak width (2D)	15
Minimum signal/noise (S/N)	25
Segmented Processing S/N	250
Number of hits to return	5
Minimum mol Weight	45
Maximum mol Weight	500
Mass Threshold	10
Minimum similarity match	600
**Stat Compare options**	
ChromaTof version	V4.50
Minimum similariy match	700
Maximum modulation periods apart	1
Maximum RT difference (s)	0.1
Minimum number of samples that contain the analyte	5
Minimum percent of samples in a class that contain the analyte	50

Figure S1. OPLS-da Scores plot of the Set 1 rice varieties and the compounds with VIP scores greater than 1.3.

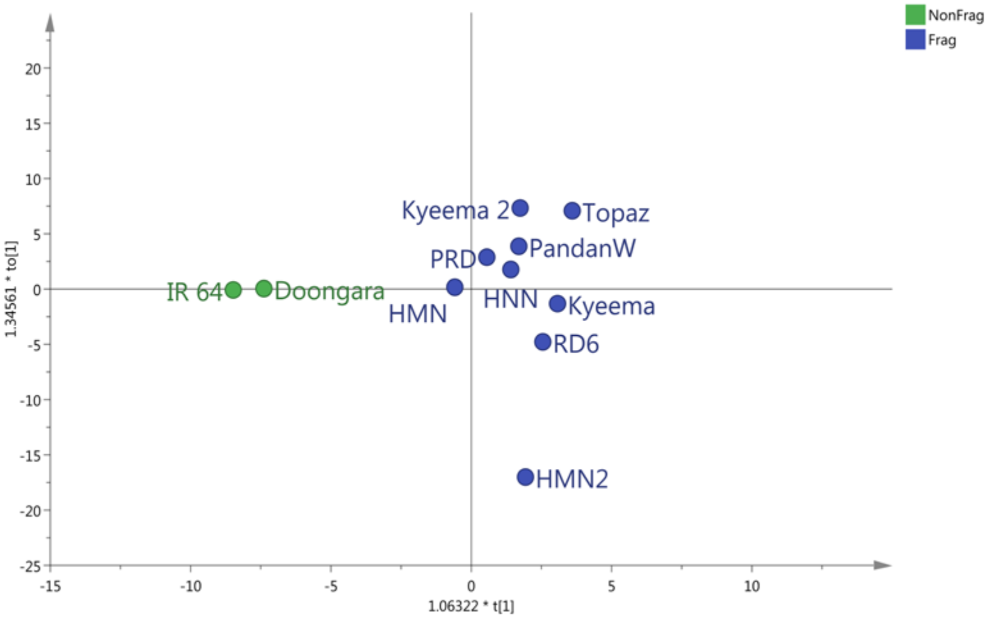

fragrant rice		nonfragrant rice	
**Putative compound**	**VIP**	**Putative compound**	**VIP**
Pyrrole	2.2144	Decane	1.8499
2-Acetyl-1-pyrroline	1.6191	1-Octene, 3,7-dimethyl-	1.83784
Analyte 5	1.60788	Benzene, 1,2,3-trimethyl-	1.79628
Ethanone, 1-(1H-pyrrol-2-yl)-	1.47624	Hydroperoxide, 1-methyl-1-phenylethyl	1.73047
Analyte 4	1.44464	2-Butanone	1.71
1-Pentene, 2,3,3-trimethyl-	1.33509	Benzene, 1-ethyl-3-methyl-	1.69128
2-Decen-1-ol	1.32913	Heptane, 2,2,4,6,6-pentamethyl-	1.6274
Pentanal	1.3135	2-Butanol	1.62546
		Toluene	1.62535
		Indole	1.56015
		Octane	1.38539
		Propane, 2-ethoxy-2-methyl-	1.3427
		1-Pentanol	1.33878

Figure S2. Mass spectral EI pattern of chemical standards analysed by GC × GC-TOF-MS (A) 2-acetylpyrrole (RT: 820, 2.12s) *m/z* (%): 109 (60), 95 (6), 94 (100), 80 (2), 66 (80), 65 (8), 53 (11), 50 (7), 40 (18), 39 (60), 38 (23), 37 (10), 22 (22); (B); 2-acetyl-1-pyrroline (RT: 627.5, 1.02s) *m/z* (%): 83 (22), 69 (8), 68 (10), 43 (100), 41 (50), 42 (20), 39 (16); (C) pyrrole (RT:475, 2.25s) *m/z* (%): 68 (4), 67 (100), 66 (8), 39 (83), 41 (72), 40 (55), 38 (35), 37 (23), 52 (4).

A

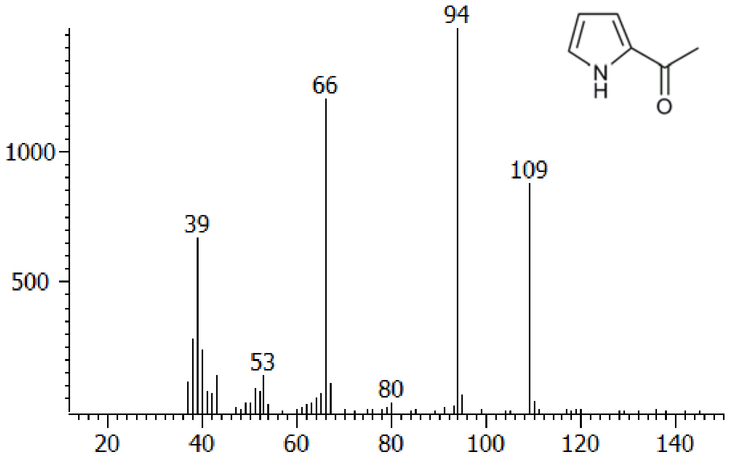

B

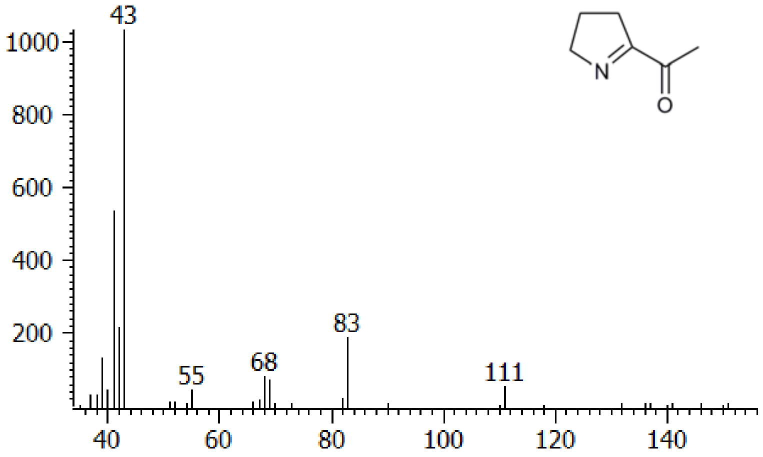

C

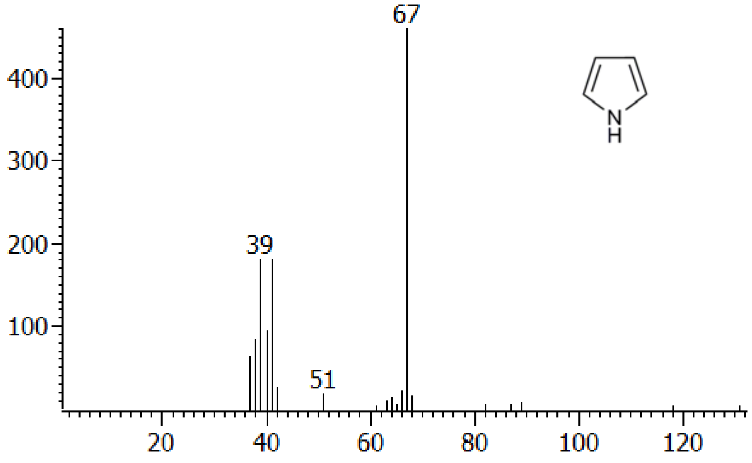

Figure S3. GC × GC HRT 4D EI spectra of (A) Analyte 4 (1-pyrroline), and (B) Analyte 5 (6M5OTP).

A

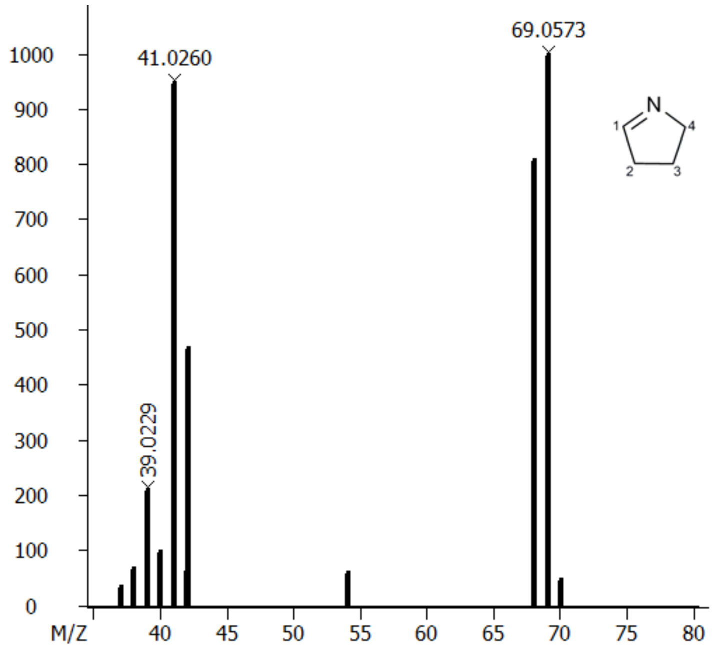

B

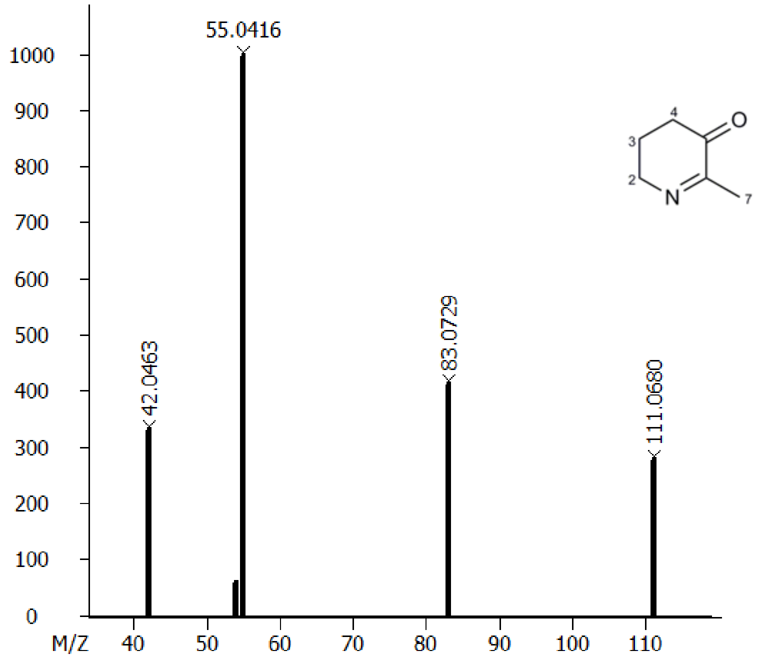

Figure S4. Identification of Analyte 4 (1-pyrroline). MS Electron ionisation fragmentation pattern of Analyte 4 detected in GC × GC-TOF-MS (A) rice, and (B) synthesised 1-pyrroline standard. (C) ^{1}H NMR trace of the synthesised 1-pyrroline standard.

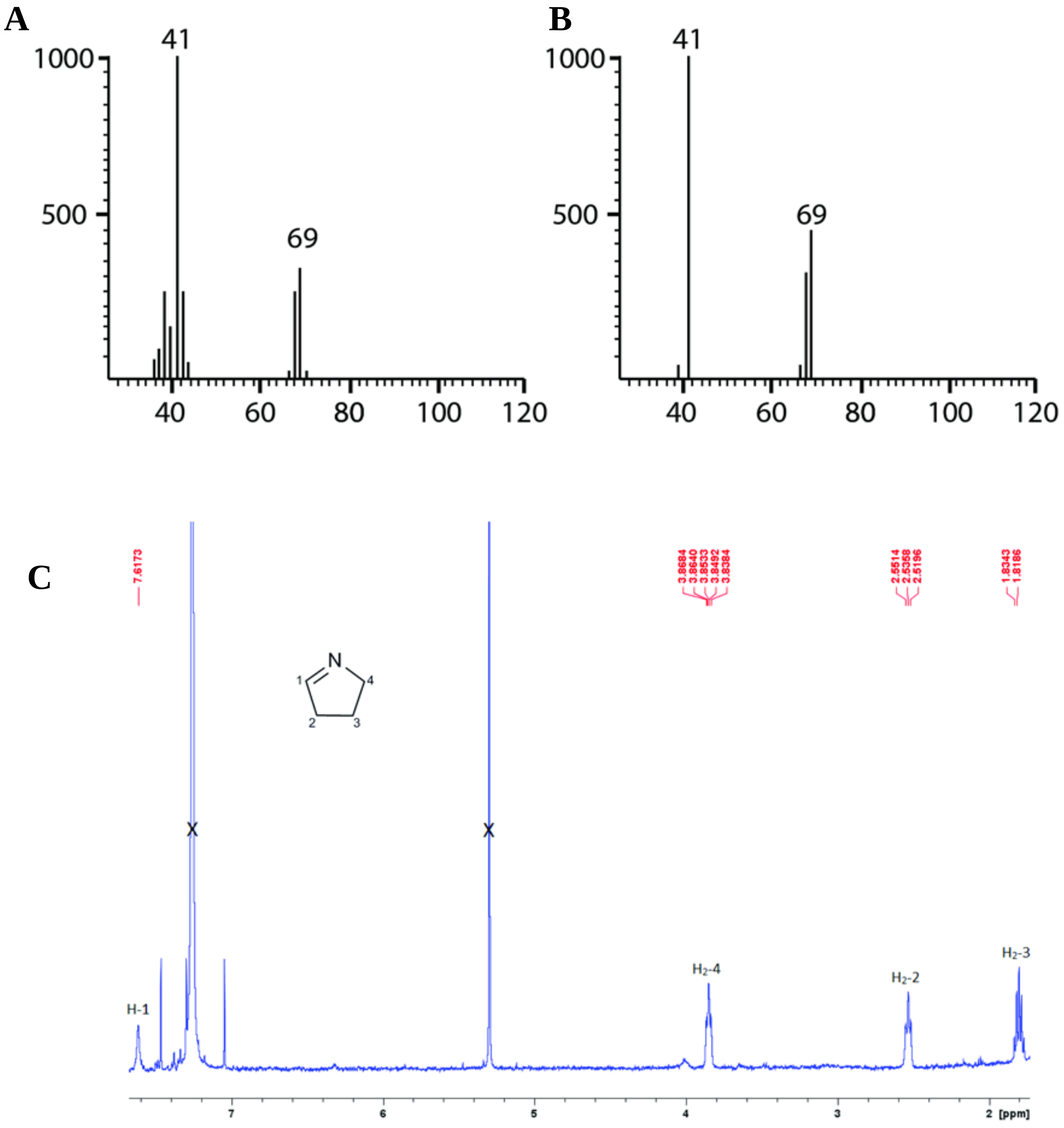

Figure S5. (A) HSQC and (B) HMBC of 2AP and 6M5OP.

A

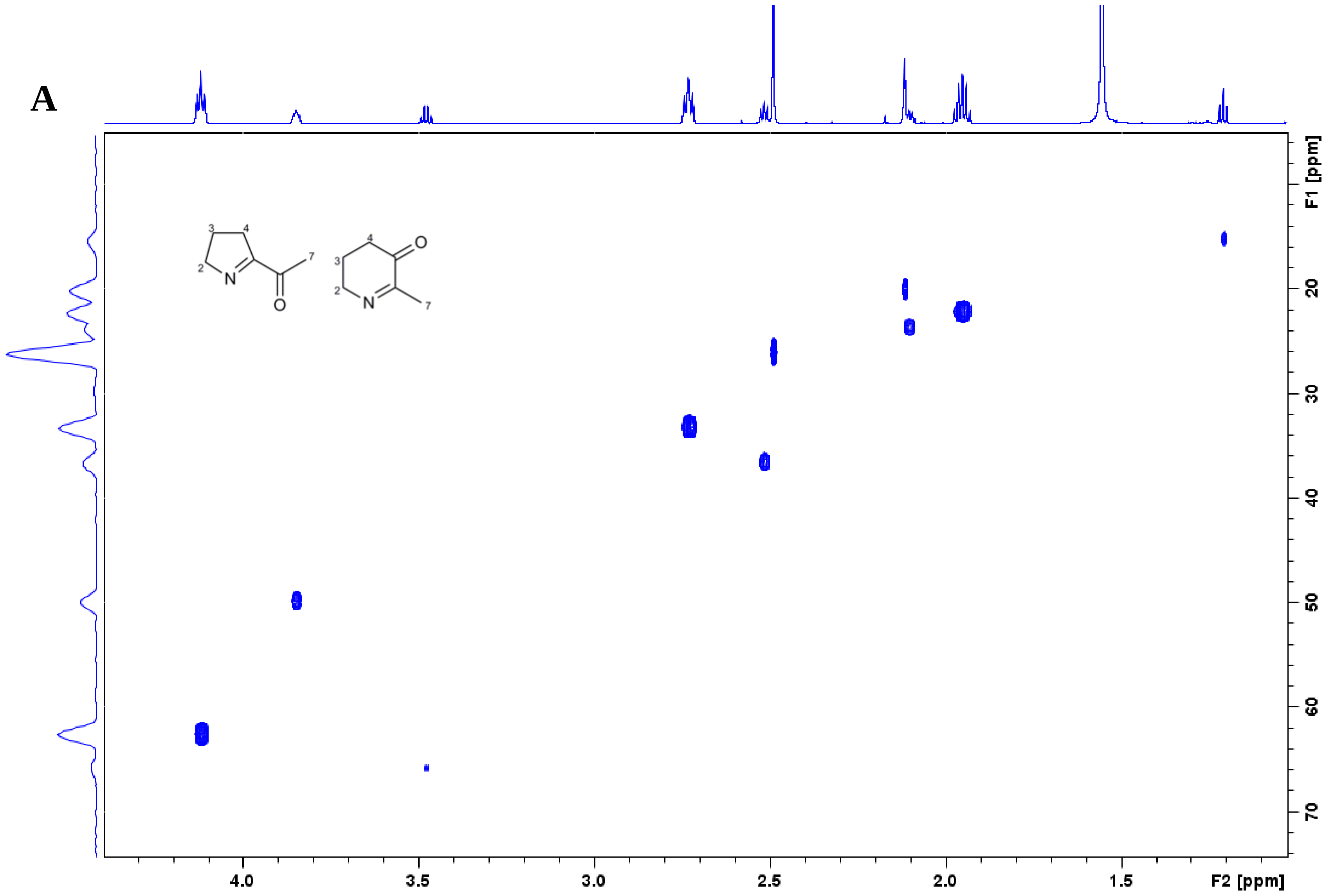

B

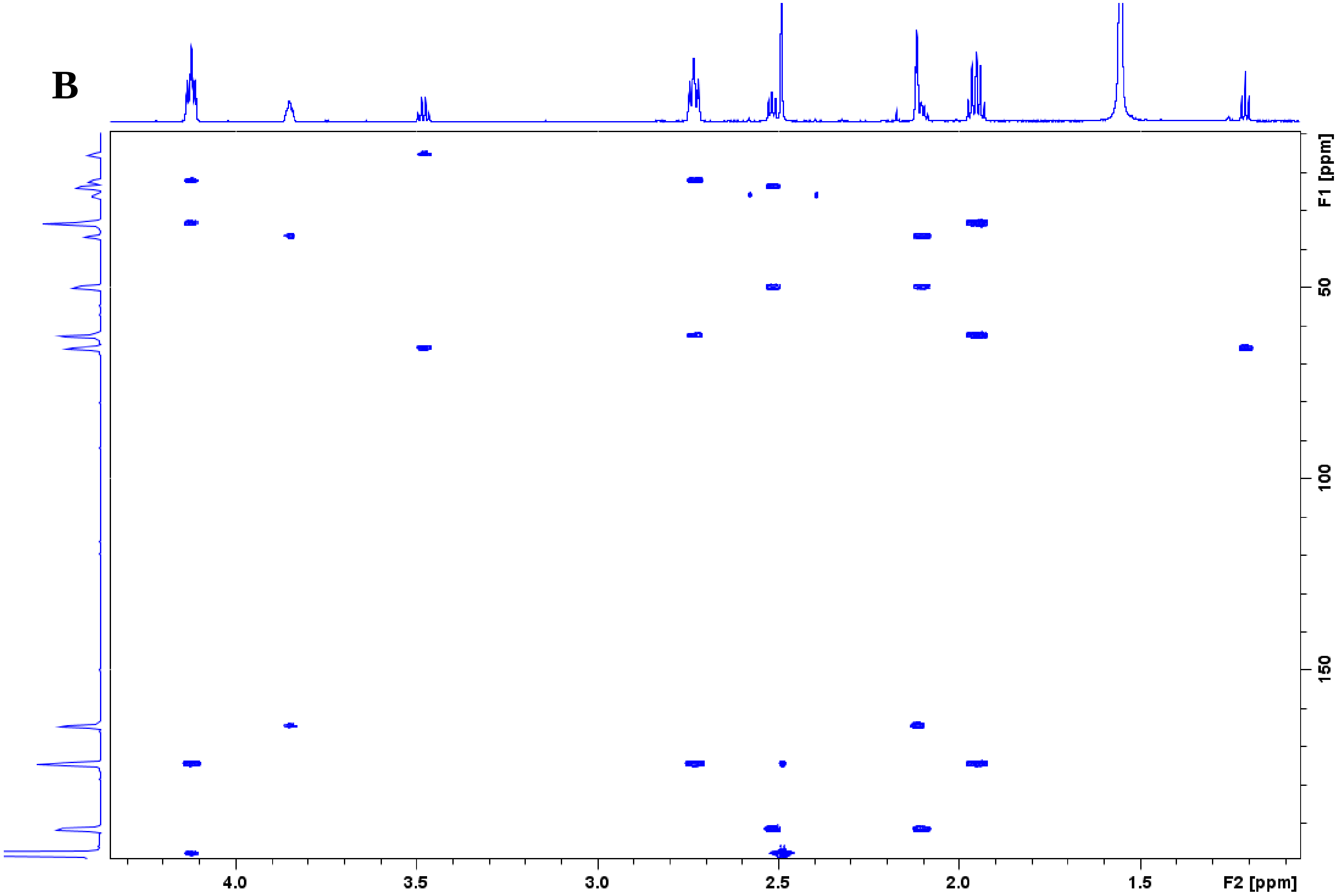

Figure S6. Manhattan plots for 2-AP (A); pyrrole (B); 1-pyrroline (Analyte 4) (C); 6M5OTP (Analyte 5) (D); 2-acetylpyrrole (E). QQ plot for 2AP (pink), pyrrole (yellow), 1-pyrroline (Analyte 4) (green), 6M5OTP (red), 2-accetylpyrrole (blue) (F) before addition of the *FGR* functional allele in the SNP matrix.

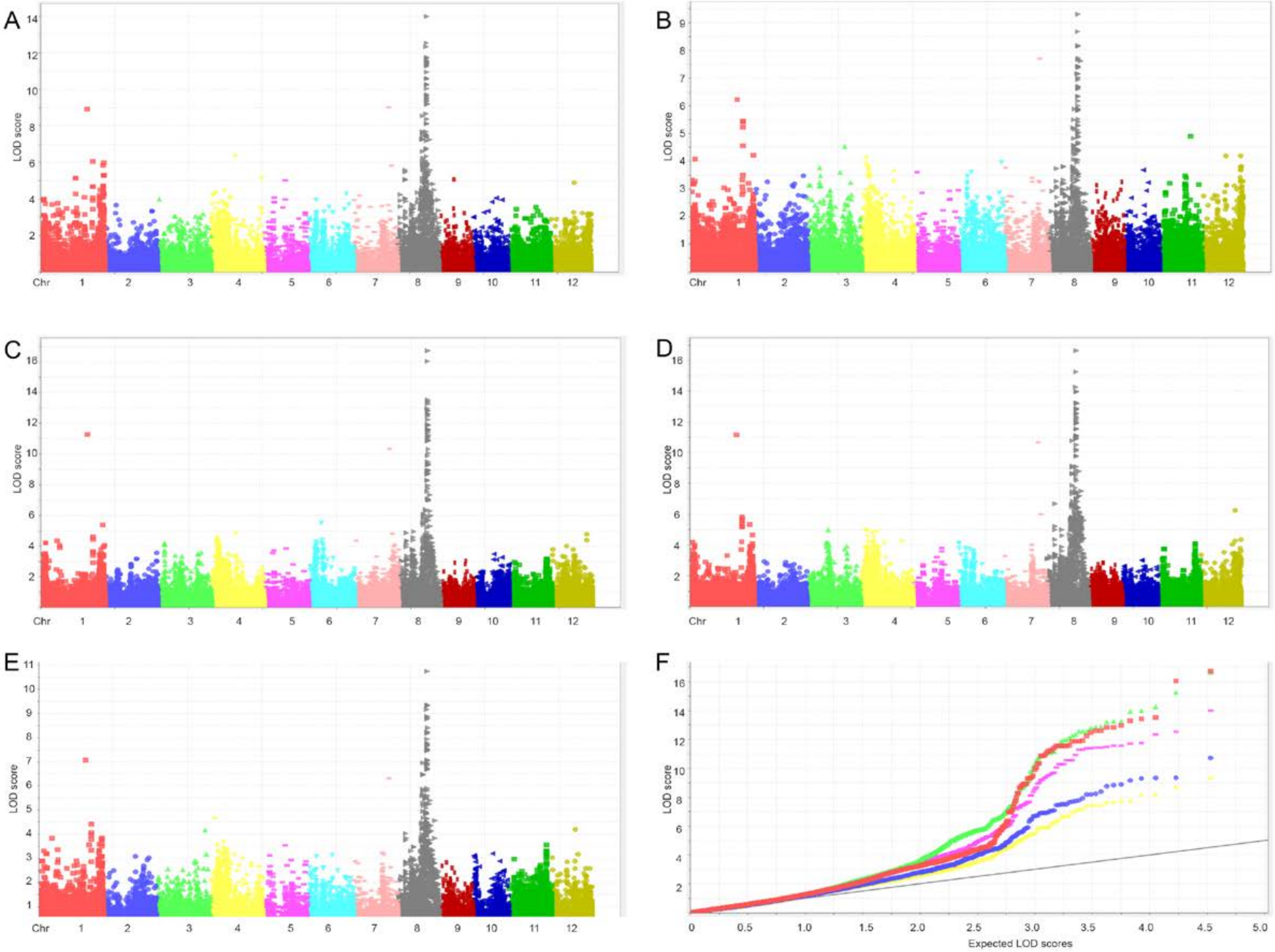

Supplement: Supplementary file 1 — Supplementary Information [file 41598_2017_7693_MOESM1_ESM.pdf]
